# Simultaneously estimating food web connectance and structure with uncertainty

**DOI:** 10.1002/ece3.8643

**Published:** 2022-03-08

**Authors:** Anubhav Gupta, Reinhard Furrer, Owen L. Petchey

**Affiliations:** ^1^ 27217 Department of Evolutionary Biology and Environmental Studies University of Zurich Zurich Switzerland; ^2^ Department of Mathematics and Department of Computational Science University of Zurich Zurich Switzerland

**Keywords:** ABC, ADBM, connectance, food web, true skill statistic, uncertainty

## Abstract

1. Food web models explain and predict the trophic interactions in a food web, and they can infer missing interactions among the organisms. The allometric diet breadth model (ADBM) is a food web model based on the foraging theory. In the ADBM, the foraging parameters are allometrically scaled to body sizes of predators and prey. In Petchey et al. (*Proceedings of the National Academy of Sciences*, 2008; 105: 4191), the parameterization of the ADBM had two limitations: (a) the model parameters were point estimates and (b) food web connectance was not estimated.
2. The novelty of our current approach is: (a) We consider multiple predictions from the ADBM by parameterizing it with approximate Bayesian computation, to estimate parameter distributions and not point estimates. (b) Connectance emerges from the parameterization, by measuring model fit using the true skill statistic, which takes into account prediction of both the presences and absences of links.
3. We fit the ADBM using approximate Bayesian computation to 12 observed food webs from a wide variety of ecosystems. Estimated connectance was consistently greater than previously found. In some of the food webs, considerable variation in estimated parameter distributions occurred and resulted in considerable variation (i.e., uncertainty) in predicted food web structure.
4. These results lend weight to the possibility that the observed food web data is missing some trophic links that do actually occur. It also seems likely that the ADBM likely predicts some links that do not exist. The latter could be addressed by accounting in the ADBM for additional traits other than body size. Further work could also address the significance of uncertainty in parameter estimates for predicted food web responses to environmental change.

## Graphical abstract

To date, food web models have not simultaneously predicted structure and connectance. Using approximate Bayesian computation, both the structure and complexity (connectance) of a food web model is estimated. Novel insights include that connectance is almost always overestimated, with implications for model and data improvement.

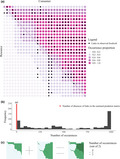

## 1 INTRODUCTION

Knowledge about the trophic interactions among the organisms in a community is crucial for understanding the structure and dynamics of ecological communities and for predicting their response to environmental change (Bergamino et al., 2011; Dunne et al., 2002; Krause et al., 2003; Lurgi et al., 2012; Morris et al., 2015; O'Connor et al., 2009; Tylianakis & Binzer, 2014). The network of trophic interactions is often referred to as a food web. The food web structure can provide answers to key ecological questions: which species are more vulnerable to environmental changes such as temperature (Petchey et al., 1999); how robust a food web is to extinctions (Dunne et al., 2002); and how a food web reacts if the predators are removed (Knight et al., 2005)?

Trophic interactions information from multiple sources can be used to infer a food web, for example, gut contents (Peralta‐Maraver et al., 2016) and cannot be assigned with certainty to a specific prey item (Baker et al., 2014). With stable isotope ratios of tissues, uncertainty may be due to factors such as variability in the isotopic fractionation values across multiple combinations of diets and tissues/species, unquantified temporal, or spatial variation in prey isotopic values, and variation caused by routing of particular dietary nutrients into particular tissues (Crawford et al., 2008). Furthermore, complete recording of all interactions usually requires a large sampling effort even at small spatial and temporal scales (Hobson et al., 1994). Food web structure is very difficult to record at larger spatial and temporal scales without losing resolution (spatial, temporal, and taxonomic; Gravel et al., 2013; Jord'an & Osváth, 2009; Martinez, 1991). Less than complete sampling of interactions can result in no interaction being observed between a pair of individuals that in fact do interact, which results in missing links in a food web. Due to under‐sampling, food webs can be poorly understood, which may hinder further advances in the field (Martinez et al., 1999).

When interactions are difficult to observe, and hence, well‐documented food webs are not available, models which predict species interactions may provide a solution (Allesina et al., 2008; Cohen et al., 1985; Gravel et al., 2013; Petchey et al., 2008; Tamaddoni‐Nezhad et al., 2013). A food web model can be used to predict missing information about species interactions. For example, Petchey et al. (2008) showed how a model of species interactions (and therefore food web structure) could be parameterized from data on the known presence and absence of trophic interactions. The model and its parameter values encode the rules for occurrence or absence of species interactions to predict food web structure. Observed data may be used to select and parameterize the model. Tamaddoni‐Nezhad et al. (2013) used large agricultural datasets, logic‐based machine learning, and text mining to assign interactions between nodes to automatically construct food webs. Gravel et al. (2013), inspired by the niche model of food web structure, developed a method that used the statistical relationship between predator and prey body size to infer the food web.

Food web models are also useful for ecological forecasting. Lindegren et al. (2010) used a stochastic food web model driven by regional climate scenarios to produce quantitative forecasts of cod dynamics in the twenty‐first century. Hattab et al. (2016) forecasted the potential impacts of climate change on the local food web structure of the highly threatened Gulf of Gabes ecosystem, located in the south of the Mediterranean Sea. Hence, food web models have an important role in filling gaps in knowledge about species interactions, including predicting future changes in food web structure.

The allometric diet breadth model (ADBM) was the first model able to predict food web connectance (i.e., the number of realized trophic links divided by the number of potential links) and structure (i.e., the arrangement of trophic links; Beckerman et al., 2006; Petchey et al., 2008). Models such as the Cascade model require connectance as an input parameter, whereas the ADBM does not (Cohen et al., 1985). It uses foraging theory, specifically the contingency model (MacArthur & Pianka, 1966), to predict the diet of each potential consumer and thereby the food web structure (further details are in the Material and Methods section). The ADBM had variable success in explaining the structure of 15 different food webs, with the proportion of links correctly predicted ranging from 7% to 54% (Table 1). The ADBM correctly predicted 54% of the presence of links in the Benguela Pelagic food web. The poorest prediction of presence of links was for the Grasslands food web with only 7% of the presence of links correctly predicted. When trophic interactions were more strongly dependent on size, the model correctly predicted a greater proportion of links. Indeed, constructing a food web based only on body size (i.e., ignoring taxonomy) resulted in almost twice the number of correctly predicted links, that is, 83%, in contrast to taxonomy (Woodward et al., 2010).

**TABLE 1:** Information about the food webs predicted using the ADBM

Common food web name (Original Publication)	Predation matrix source	Body size source	General ecosystem	Number of species	Connectance	Body size range (approximate)	Proportion of presence of links correct	Type of interactions
Benguela Pelagic (Yodzis, 1998)	Brose et al. (2005)	Brose et al. (2005)	Marine	30	0.21	$10^{‐8}$ to $10^{6}$	0.54	Predation
Broadstone Stream (taxonomic aggregation) (Woodward & Hildrew, 2001; Woodward et al., 2005)	Brose et al. (2005)	Brose et al. (2005)	Freshwater	29	0.19	$10^{‐6}$ to $10^{‐2}$	0.40	Predation
Broom (Memmott et al., 2000)	Brose et al. (2005)	Brose et al. (2005)	Terrestrial	60	0.03	$10^{‐6}$ to $10^{0}$	0.09	Herbivory, Parasitism, Predation, Pathogenic
Capinteria (Lafferty et al., 2006)	Hechinger et al. (2011)	Hechinger et al. (2011)	Marine (Salt Marsh)	88	0.08	$10^{‐6}$ to $10^{4}$	0.33	Predator‐parasite, Parasite‐parasite
Caricaie Lakes (Cattin et al., 2004)	Brose et al. (2005)	Brose et al. (2005)	Freshwater	158	0.05	$10^{‐5}$ to $10^{5}$	0.13	Predation, Parasitism
Grasslands (Dawah et al., 1995)	Brose et al. (2005)	Brose et al. (2005)	Terrestrial	65	0.03	$10^{‐3}$ to $10^{‐2}$	0.07	Herbivory, Parasitism
Mill Stream (Ledger, Edwards, Woodward unpublished)	Brose et al. (2005)	Brose et al. (2005)	Freshwater	80	0.06	$10^{‐6}$ to $10^{‐2}$	0.36	Herbivory, Predation
Skipwith Pond (Warren, 1989)	Brose et al. (2005)	Brose et al. (2005)	Freshwater	71	0.07	$10^{‐4}$ to $10^{‐1}$	0.14	Predation
Small Reef (Opitz, 1996, table 8.6.2)	Cirtwill and Eklöf (2018)	Cirtwill and Eklöf (2018)	Marine (Reef)	239	0.06	$10^{‐11}$ to $10^{5}$	0.30	Predation, Herbivory
Tuesday Lake (Jonsson et al., 2005)	Brose et al. (2005)	Brose et al. (2005)	Freshwater	73	0.08	$10^{‐11}$ to $10^{3}$	0.46	Predation
Ythan (Emmerson & Raffaelli, 2004)	Cirtwill and Eklöf (2018)	Cirtwill and Eklöf (2018)	Marine (Estuarine)	85	0.04	$10^{‐6}$ to $10^{4}$	0.17	Predation
Broadstone Stream (size aggregation) (Woodward et al., 2010)	Woodward (2021)	Woodward (2021)	Freshwater	29	0.24	$10^{‐7}$ to $10^{2}$	0.83	Predation

Although Petchey et al. (2008) demonstrated that foraging theory could predict food web structure, their implementation of the ADBM included at least two limitations. The parameterization method provided estimates of the parameters with no uncertainty: A single set of parameter values that maximized the explanatory power was selected. In other words, the parameterization method led to point estimates of the parameters that predicted a single food web structure (because the ADBM is purely deterministic). Moreover, the best predicted food web was not exactly the same as the observed one. In a sense then, the parameterization method used in Petchey et al. (2008) was akin to estimating the intercept and slope of a regression line, but not any uncertainty in those parameters. Given that uncertainty is an essential dimension in ecological models, and in predictions about the future state of ecological communities (Carpenter, 2016; Petchey et al., 2015), this is an important limitation.

The second limitation was that the connectance of the predicted food web was not estimated. Although the ADBM can in principle estimate connectance, Petchey et al. (2008) prevented the model from doing so. They set the value of relevant parameters in the model to instead ensure the predicted connectance was equal to the observed connectance. Moreover, fixing predicted connectance to be equal to observed connectance does not account for the possibility that the observed connectance was imperfectly measured. Indeed, if low effort was used to observe the trophic links in a community, the observed connectance are likely to be lower than if all trophic links were observed. Connectance is an important driver for the stability and dynamics of a food web (May, 1972) and most of the structural properties of food webs co‐vary with connectance (Dunne et al., 2002; Poisot & Gravel, 2014); thus, this limitation must be addressed.

In this article, we report on how we address these limitations. We removed the first limitation by applying approximate Bayesian computation (ABC). The approach originated in population genetics and has been used in a wide range of research fields: systems biology (Toni et al., 2009), ecology (Jabot & Chave, 2009), epidemiology (Shriner et al., 2006), and ecological networks (Ibanez, 2012; Poisot & Stouffer, 2016). One of the advantages of ABC is that it does not require a likelihood function. As ADBM is a complex deterministic model where the likelihood cannot be explicitly evaluated, ABC is a good choice of parameterization.

We addressed the second limitation by allowing estimation of number of links as well as arrangement of links. To accomplish this, we measured model fit by using the true skill statistic (TSS), which takes into account both the number of presences and absences of links correctly predicted (see section *Choice of distance measure* for definition of the TSS). High values of the TSS occur when both the predicted arrangement of links and the predicted number of links are close to the observed arrangement and number of links, respectively.

## 2 MATERIALS AND METHODS

In the upcoming sections, we present a detailed account of the application of ABC to parameterize the ADBM, the description of the ADBM and of the food web data we used. We explain the rejection Monte Carlo ABC method in the main text and Markov chain Monte Carlo ABC and sequential Monte Carlo ABC methods in the Supplementary information (hereafter SI) Section S1 (hereafter SI‐S1). We computed accuracy with the TSS to assess the ADBM's predictions and calculated different food web properties to compare these predictions across food webs.

### 2.1 Allometric Diet Breadth Model (ADBM)

The allometric diet breadth model (ADBM) is based on optimal foraging theory, specifically the contingency foraging model (MacArthur & Pianka, 1966). The ADBM predicts the set of prey species a consumer should feed upon to maximize its rate of energy intake (Petchey et al., 2008; hereafter referred as PBRW study). The species in this set are assumed to have the trophic link with the predator. To make these predictions, the model assumes that a foraging predator is in one of two exclusive states: searching for prey or handling a prey item. The notation used below corresponds to that of (Petchey et al., 2008). The model requires four variables for each potential predator–prey interaction:

- The energy content of the resources $E_{i}$ (only prey i specific) (energy).
- The handling times $H_{\mathrm{ij}}$, which is the time not spent searching caused by consuming a prey item (prey i and predator j specific) (time).
- The space clearance rates $A_{\mathrm{ij}}$ (also known as the attack rate; prey i and predator j specific) (area or volume per time).
- The prey densities $N_{i}$ (only prey i specific) (individuals per area or volume).

The term “Allometric” in the ADBM refers to the use of four allometric relationships, one for each of these four variables, including the body size of the predator $M_{j}$ and prey $M_{i}$ (Table 2). With these four allometric relationships, and the body size of each of the species in a community, we can predict the four variables (energy, handling time, space clearance rate, and prey density) and then use the contingency foraging model to predict diets.

**TABLE 2:** Traits with their allometric function and corresponding parameters in ADBM

Traits (Unit)	Allometric function	Parameters	Comments
Energy $\left(\text{Joules}\right)$	$E_{i}=eM_{i}$	e	Arbitrary. No effect on structure^a^
Abundance $\left(\text{individual}/m^{2}\;\text{or}\;\text{individual}/m^{3}\right)$	$N_{i}=nM_{i}^{n_{i}}$	n	Connectance affected by the product nah ^a^
		$n_{i}$	Assumed value of $\frac{‐3}{4}$ based on empirical data
Space Clearance Rate $\left(m^{2}/s\;\text{or}\;m^{3}/s\right)$	$A_{\mathrm{ij}}=aM_{i}^{a_{i}}M_{j}^{a_{j}}$	a	Connectance affected by the product nah ^a^
			Estimated using ABC
		$a_{i}$	Estimated using ABC
		$a_{j}$	Estimated using ABC
Handling time $\left(s\right)$	$H_{\mathrm{ij}}=\frac{h}{b‐\frac{M_{i}}{M_{j}}}\;\text{if}\;\frac{M_{i}}{M_{j}}<b$ $H_{\mathrm{ij}}=\infty \;\text{if}\;\frac{M_{i}}{M_{j}}\geq b$	h	Connectance affected by the product nah ^a^
		b	Estimated using ABC

^a^ See SI S5 for further explanation for why only four parameters were estimated.

Each of the four allometric equations has parameters: a constant and/or at least one exponent (Table 2). It is the value of some of these parameters that can be estimated to have the predicted food web structure match (as closely as possible) the structure of an observed food web. This is akin to choosing values of slope and intercept of a linear regression that maximizes the fit of the regression line to the observed data.

Please note that in the notation of the allometric exponents ($a_{i}$, $a_{j}$), the i and j refer to the exponent for the prey mass and predator mass, respectively, that is, the i and j refers to the role and are not species specific.

Because some of the allometric constants and exponents are known, and because others are redundant with respect to each other (see Table 2 for details), we estimate only the following parameters: a, $a_{i}$, $a_{j}$ and b in the model (Table 2).

In the ADBM, some species can be predicted to eat others, but to not be eaten by others, that is, be predicted to be a top predator. This can occur for relatively large species when the exponent b is less than 1, which can cause the handling time of this large species to be infinite for all potential consumer species.

### 2.2 Observed food web data

The observed food webs that we fit the ADBM to belong to marine, freshwater, and terrestrial ecosystems (Table 1). The observed connectance of these food webs is from 0.03 to 0.24 and there are 29 to 239 species. The food webs contain primary producers, herbivores, carnivores, parasites, and parasitoids. They also contain various types of feeding interactions, including predation, herbivory, bacterivory, parasitism, pathogenic, and parasitoid.

The goodness of fit of the ADBM's predictions depends on the types of interactions in the food webs in the PBRW study. Specifically, predictions of interactions that are more size structured, such as predacious and aquatic herbivory interactions were predicted better than less size structured ones, for example, parasitoid and terrestrial herbivory ones.

All food webs with one exception (Broadstone Stream) were available only at the species level, that is, with information about interactions between species and the body size of species. We use the term “species” in this study to indicate a “nodeh” in a food web in which nodes are connected by trophic interactions, and nodes are a collection of individuals that share links. These species/nodes are not always taxonomic species, but can be broader taxonomic ranks.

In contrast, the Broadstone Stream food web data contained interactions between individuals and the individual body sizes. Thus, the Broadstone Stream food web can be constructed by aggregating by either taxonomy or size (Woodward et al., 2010).

### 2.3 Parameter estimation: Approximate Bayesian Computation

**FIGURE 1:**
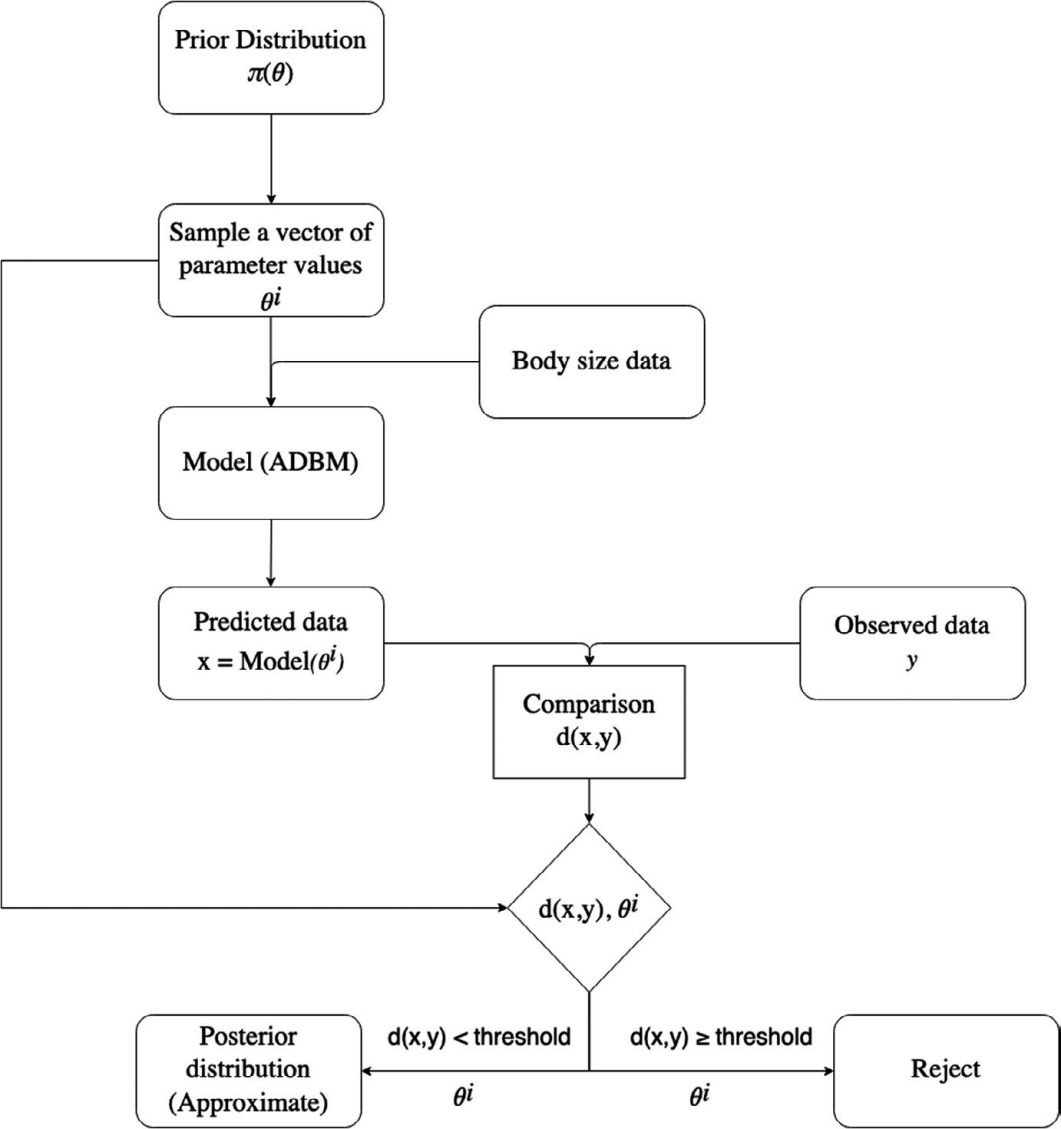
Flowchart of rejection approximate Bayesian computation method implemented to parameterize the ADBM

We used approximate Bayesian computation (ABC) to identify sets of parameter values that resulted in predicted food webs that were close in structure to the observed food web. ABC is an approach that does not require a likelihood function. Instead, there is a distance function that measures the distance between a model's prediction and the observed data. The approximation of the likelihood depends on the ABC method used, which is further discussed below and SI. The model parameter values are sampled from a prior distribution. The accepted parameter values form an approximate posterior distribution for the model parameter. We implemented three ABC methods to parameterize the ADBM: namely rejection Monte Carlo (Figure 1), Markov chain Monte Carlo, and sequential Monte Carlo. The three methods produced very similar results (Figures S26 and S27), and we therefore only include the simplest (rejection) in this main text.

#### 2.3.1 Prior distribution

The prior distributions for $a_{i}$ and $a_{j}$ were chosen to be uniform distributions. The range of distribution was from −1.5 to 1.5 and 0 to 3 for $a_{i}$ and $a_{j}$, respectively, informed by the estimates in Rall et al. (2012). However, we chose a prior range specific to food webs for the parameter b because body size varies greatly among the species in the observed food webs. For example, in the Benguela Pelagic food web, the body sizes of species range from the order of $10^{‐8}$ gm to $10^{5}$ gm. Hence, the range of prey–predator ratio was from the order of $10^{‐14}$ to $10^{14}$. To take this into account, we took the prior of $log_{10}\left(b\right)$ from a uniform distribution ranging from ‐15 to 15.

For the prior of a, we chose the prior of $log_{10}\left(a\right)$ to be a uniform distribution. Since the ADBM estimated connectance to be higher than the real connectance, lower values of a were favored in the parameterization. Hence, the upper bound of the prior was set to 10. To set the lower bound, we investigated how the TSS varied with $log_{10}\left(a\right)$ (e.g., Figure S28). We found that the TSS increased with decreasing $log_{10}\left(a\right)$ and then remained constant, for a constant value of $log_{10}\left(b\right)$. We therefore decided to set the lower bound of $log_{10}\left(a\right)$ such that the maximum variation of TSS was taken into account, while attempting to keep the range of prior as small as possible. In the case of Benguela Pelagic as shown in Figure S28, the lower bound of $log_{10}\left(a\right)$ was taken to be −12.

#### 2.3.2 Comparison of observed and predicted

The difference between the model's prediction and the observed data (e.g., the sum of squared residuals is such a distance in linear regression) is quantified by a distance measure. The distance is lower when there is a closer match between the model's prediction and the observation. A perfect match would result in zero distance.

The magnitude of the distance is used for the acceptance or rejection of a set of parameter values. An accepted set of parameter values contributes to the posterior distribution, rejected ones do not. This makes the distance measure one of the important features of ABC. A threshold distance is chosen, and if the distance for a particular set of parameter values is less than the threshold, then that set of parameter values contributes to the posterior distribution. When the distance is greater than the threshold, the parameter values do not contribute to the posterior. Hence, the magnitude of the distance threshold determines the proportion of a model's parameters that are accepted. A higher threshold causes a high proportion of acceptances but less accuracy with the acceptance of some parameter sets that result in predictions quite unlike the observed data. Below, we first describe and justify our choice of distance measure and then our choice of threshold.

##### Choice of distance measure

In the PBRW study, the measure of distance was equivalent to $1‐TP/\left(TP+FN\right)$, where TP is the number of observed links that were predicted (the number of true positives) and FN is the number of observed links that were not predicted (the number of false negatives). A distance of 0 indicates that all observed links were correctly predicted. One way for the ADBM to achieve this is to predict that every species has a trophic link with every other species including itself—a fully connected food web with connectance of 1. The PBRW study prevented this by constraining the number of predicted links to be equal to the number of observed links, that is, the model connectance was fixed to be the same as the observed connectance. In this study, we relaxed this constraint, with the number of links as well as the arrangement of links being estimated. The first step was to choose an appropriate distance measure.

The distance measure used in this study is 1 minus the true skill statistic (TSS): distance=1‐TSS. This distance ranges from 0 to 2.

TSS is defined as:

$$\text{TSS}=\frac{TP\cdot TN‐FP\cdot FN}{\left(TP+FN\right)\left(FP+TN\right)}$$

where TP is the number of observed links that are predicted by the model (true positives), TN is the number of observed absences of links that are correctly predicted (true negatives), FP is the number of false positives, and FN is the number of false negatives.

The TSS ranges from ‐1 to 1, where +1 indicates a perfect prediction. A TSS value of zero or less indicates a performance no better than random.

The inclusion of true and false negatives in the distance measure means that the best theoretically possible prediction (smallest distance) is a unique prediction, and specifically, the one in which the predicted presence and absence of links matches exactly with the observed presence and absence of links.

##### Choice of threshold value of distance

Food web dynamics and stability are strongly dependent on connectance (May, 1972), we therefore set the distance threshold (for acceptance) such that the model had a reasonable chance of predicting the observed value of connectance. Note that in the following section (*The Rejection ABC method*), we use the term tol to denote the value of the distance threshold.

**FIGURE 2:**
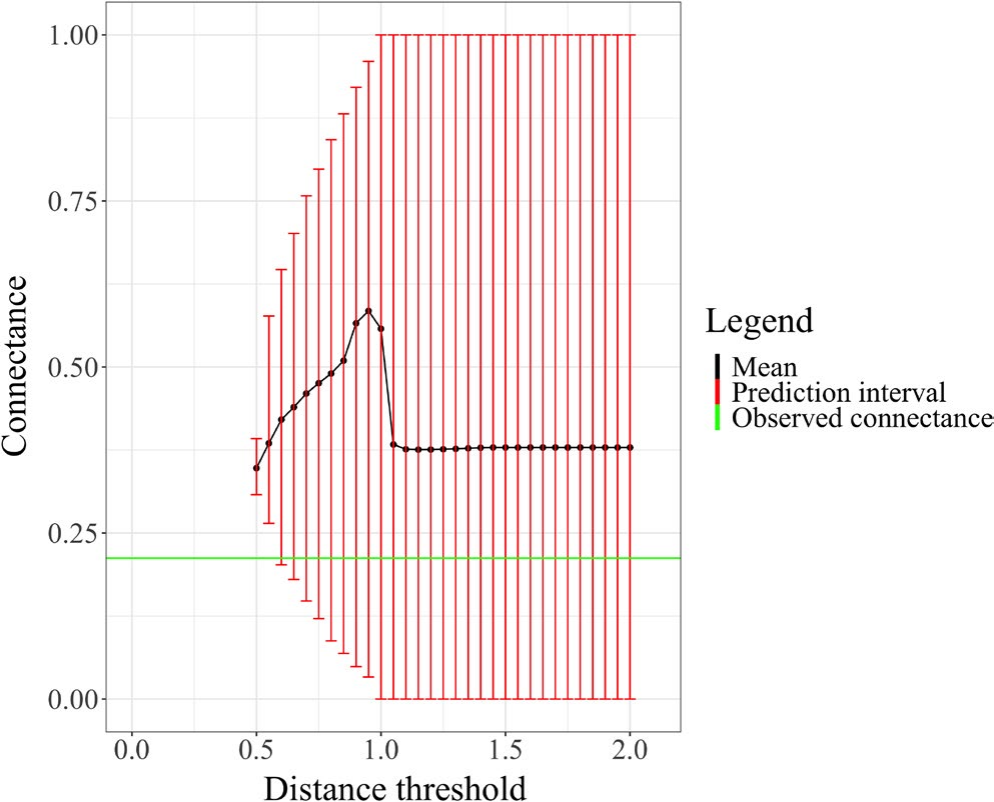
The prediction interval of the predicted connectance increases with increasing distance threshold for the Benguela Pelagic food web. The green line and black line represent the observed connectance and mean of predicted connectance, respectively

To do this, we examined how the predicted connectance varied with the distance threshold. An example of this relationship is given in Figure 2 for the Benguela Pelagic food web and in Figure S1 for other food webs. We chose the minimum threshold value that gave a range of predicted connectance containing the observed connectance.

Furthermore, it is useful to note that in Figure 2, there are no connectance values below a distance threshold value of less than 0.5 because for this particular food web, there were no sets of parameter values that achieved a better model fit than is indicated by 1‐TSS=0.5. That is, it is impossible for the ADBM to make better predictions than this. One reason for this is that the ADBM, when body size is the only trait, can only predict contiguous diets in trait space, whereas the observed data contain gaps in the diet.

#### 2.3.3 The Rejection ABC method

In the rejection ABC method, a set of parameter values are sampled from the prior distributions. This set of parameter values is either accepted and thereby added to the posterior distribution of the parameter values, or it is rejected (based on if the distance 1 − TSS is less than or greater than the threshold distance, as mentioned above). This process is repeated until there are enough acceptances to give stable (approximate) posterior distributions. Acceptance or rejection of a set of parameter values is probabilistic and depends on a weight assigned to that set of parameter values. This weight is given by a kernel function, where the weight is inversely proportional to the distance (1 − TSS). This weight is then used in a way that makes it the probability of the set of parameter values being accepted.

In the upcoming section, we further detail the rejection ABC method.

*Properties*:

- A prior distribution $\pi \left(\theta \right)$: π is the uniform distribution for parameters $\theta =\left(a,a_{i},a_{j},b\right)$
- A model prediction $model\left(\theta \right)$: ADBM $\left(\theta \right)$. This is a predicted food web, $x_{i}$, given by a particular set of parameter values $\theta_{i}$. Hence, $x_{i}=ADBM\left(\theta_{i}\right)$
- A summary statistic $s\left(x\right)$: x is the predation matrix predicted by the ADBM.
- $\begin{array}{cc} \text{A}\;\text{kernel}\;\text{function}\;\;K\left(u\right)\;:\text{epanechnikov}\;K\left(u\right) & =\frac{1}{\mathrm{tol}}\cdot \frac{3}{4}(1‐\left({\frac{u}{\mathrm{tol}}}^{2}\right)\;\;if\;\;u\leq tol\\ & =0\;\;\;\;\;\;\text{otherwise} \end{array}$

where tol is the distance threshold.

- A distance function $d\left(x_{i},y\right)$:$d\left(x_{i},y\right)=1‐TSS\left(x_{i},y\right)$
- An observed food web y, in the form of a predation matrix containing zeros and ones.

*Sampling:*

for i=1…n=1000.

- Draw a set of parameter values $\theta_{i}$ from the prior distribution $\pi \left(\theta \right)$.
- Compute the model result $x_{i}=model\left(\theta_{i}\right)$
- Compute $s\left(x_{i}\right)$ and $d\left(s\left(x_{i}\right),s\left(y\right)\right)$
- Accept or reject the parameter set probabilistically:
  Assign a weight $p_{i}$ to $\theta_{i}$ as per the kernel K; $p_{i}=\frac{K\left(d\right)}{K\left(0\right)}$, where d is the distance evaluated in the previous step.
  Compute $\alpha :U\left(0,1\right)$
  If $\alpha \leq p_{i}$, then accept $\theta_{i}$ and i=i+1

*Output:*

An approximate joint posterior distribution using the accepted $\theta_{1},\ldots ,\theta_{n}$.

### 2.4 Assessment of model fit

Accuracy is how close the model prediction is to the observation. The ADBM's prediction is a predation matrix that consists of the presence and absence of links thus comparing how close the prediction is to the observation is not as straightforward as comparing two numerical values. We defined the accuracy of the ADBM using TSS to take into account the true and false predictions of both the presence and absence of links, which is defined above.

We examined how closely structural properties of the predicted food web matched those of the observed food webs using the R *cheddar* package (Hudson et al., 2013). We evaluated properties such as proportion of basal species, proportion of intermediate species, proportion of top species, proportion of herbivores, mean omnivory, clustering coefficient, standard deviation of generality, standard deviation of vulnerability, diet similarity, mean path length, and nestedness. We could not compute mean trophic level and maximum trophic level because the networks contained too many paths for the *cheddar* package algorithm to compute.

We investigated the performance of the ADBM parameterized with the ABC by computing standardized error of the food web properties, where the standardized error is the absolute raw error (the difference between observed and predicted value) divided by the maximum absolute raw error for that property. We did not calculate the standardized error for mean omnivory and mean path length because it had some NA values and infinite values for all the food webs, respectively.

## 3 RESULTS

As an example of the model outcomes, we first present the results for the Benguela food web (e.g., predicted food web structure, variation in predicted food web structure, and posterior parameter distributions). We chose this food web as it was well explained using the method of (Petchey et al., 2008). The results of the other food webs are included in the Figures S2–S25. We then compare model outcomes across all empirical food webs between the PBRW study and our current work. We compare the TSS of the two approaches and compare some food web properties, such as proportions of basal, intermediate, and top species.

**FIGURE 3:**
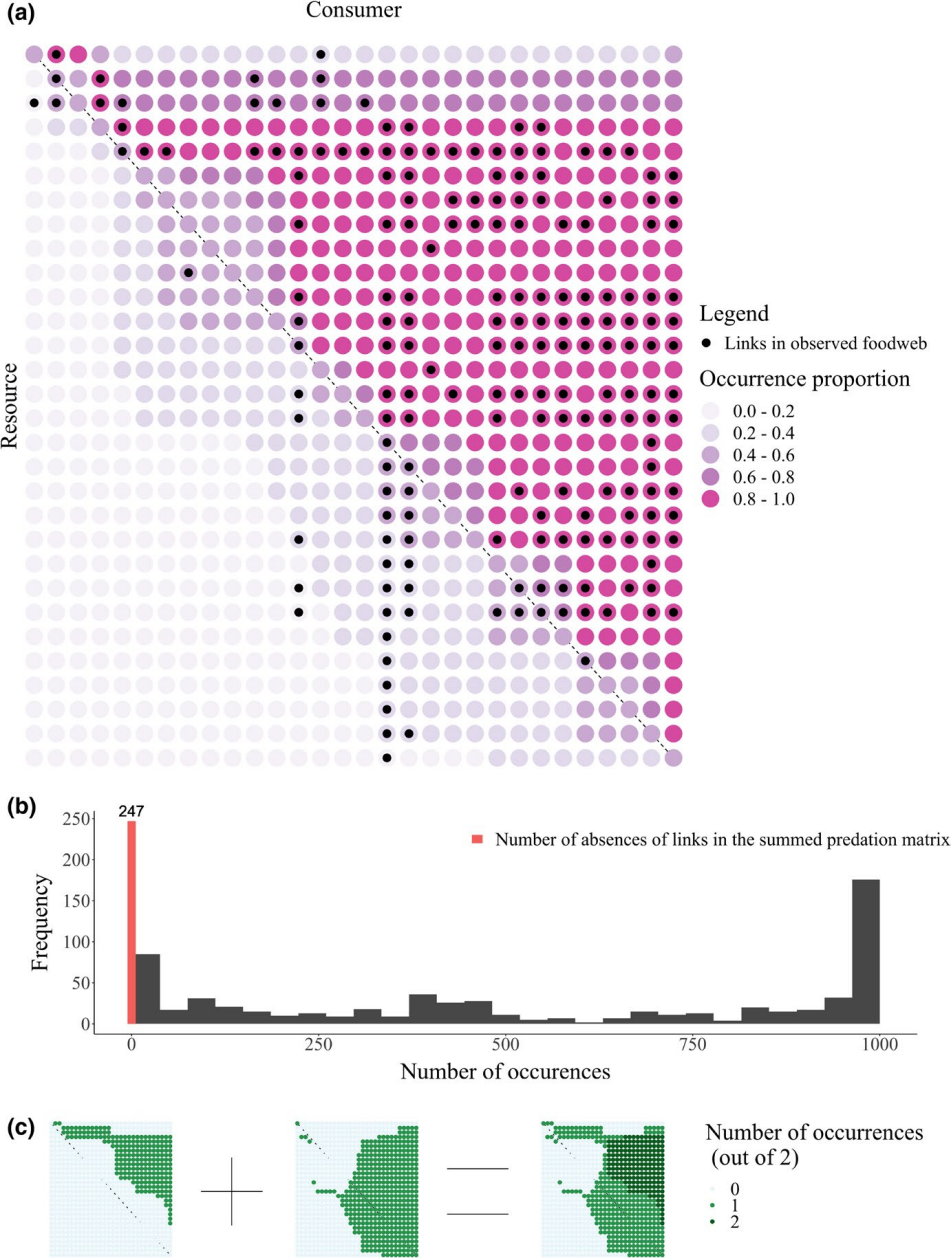
(a) Observed and predicted predation matrices for Benguela Pelagic food web. Body size increases from left to right and top to bottom along the predation matrix. Black circles show where there is an observed trophic link. The intensity of the pink circles shows the proportion of 1000 predicted food webs that had a trophic link between the corresponding species. This type of overlay is shown for two examples predicted in panel (c). (b) The histogram of the number of times a link was predicted across 1000 independently predicted food webs. There were 841 species pairs in this food web. About 150 of these were predicted to have a trophic link in all 1000 predicted predation matrices. The red bar shows the number of pairs of species for which a trophic link was never predicted. (c) Two predicted predation matrices for Benguela Pelagic food web corresponding to the minimum and the maximum value of estimated b, and their sum

The true skill statistic (TSS) of the predicted Benguela Pelagic food web varied between 0.4 and 0.52. This variation in the TSS is represented in terms of predation matrices displayed in Figure 3a, which overlays 1000 independent predation matrices accepted from the ABC method. In all the 1000 independent predation matrices, the predicted links are mostly present in the upper triangular portion of the matrix where most of the observed links are also present. Links in the upper right triangle of the predation matrix are for predators feeding on prey smaller than themselves.

**FIGURE 4:**
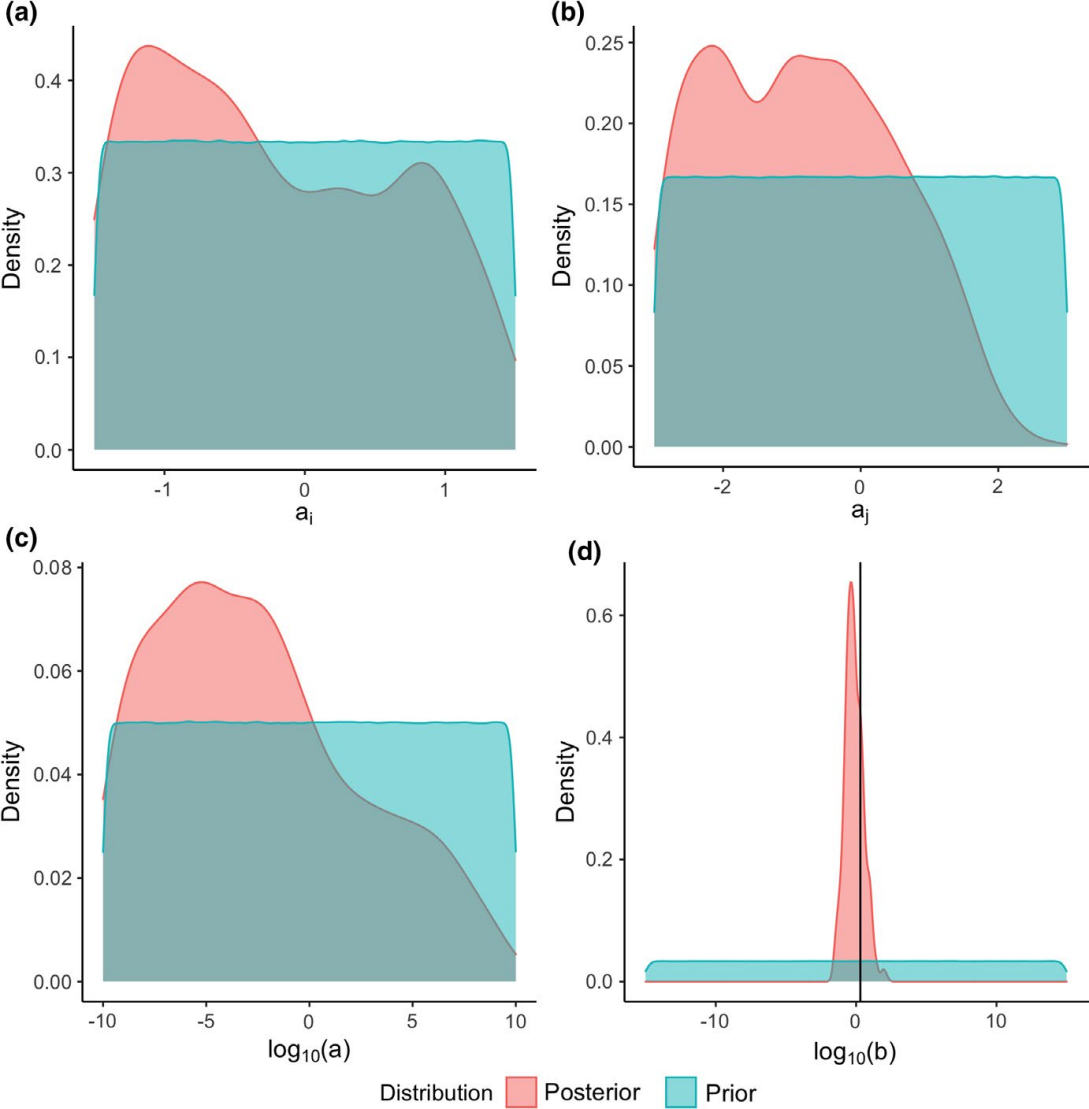
Marginal prior and marginal posterior distribution of the ADBM parameters for the Benguela Pelagic food web estimated using rejection ABC. The black vertical line in (d) corresponds to the value of b (=2) above which the most profitable prey item is larger in respect to the predator size

In the 1000 predicted predation matrices, there predators are sometimes smaller than their predicted prey, the links in the lower left triangle of the predation matrix. This is also portrayed in the marginal distribution of $log_{10}\left(b\right)$ in Figure 4d, as it includes values greater than b=2 ($log_{10}\left(b\right)=0.3$). This is relevant as values of b=2 make the most profitable prey item equal in size to the predator size. Lower values of b make the most profitable prey item smaller than the size of the predator.

There were around 250 potential links in the lower left triangle of the predation matrix that were never predicted in any of the 1000 predicted predation matrix (Figure 3b). This strongly suggests that the predator–prey size ratio of these links is so small (i.e., very large prey, very small predator) that the links cannot occur, given that the preponderance of observed links are predators consuming prey smaller than themselves.

The marginal posterior of parameter b in the Benguela Pelagic food web was more constrained than the marginal posterior distribution of the other three allometric parameters (Figure 4) as the posterior range was the narrowest.

**FIGURE 5:**
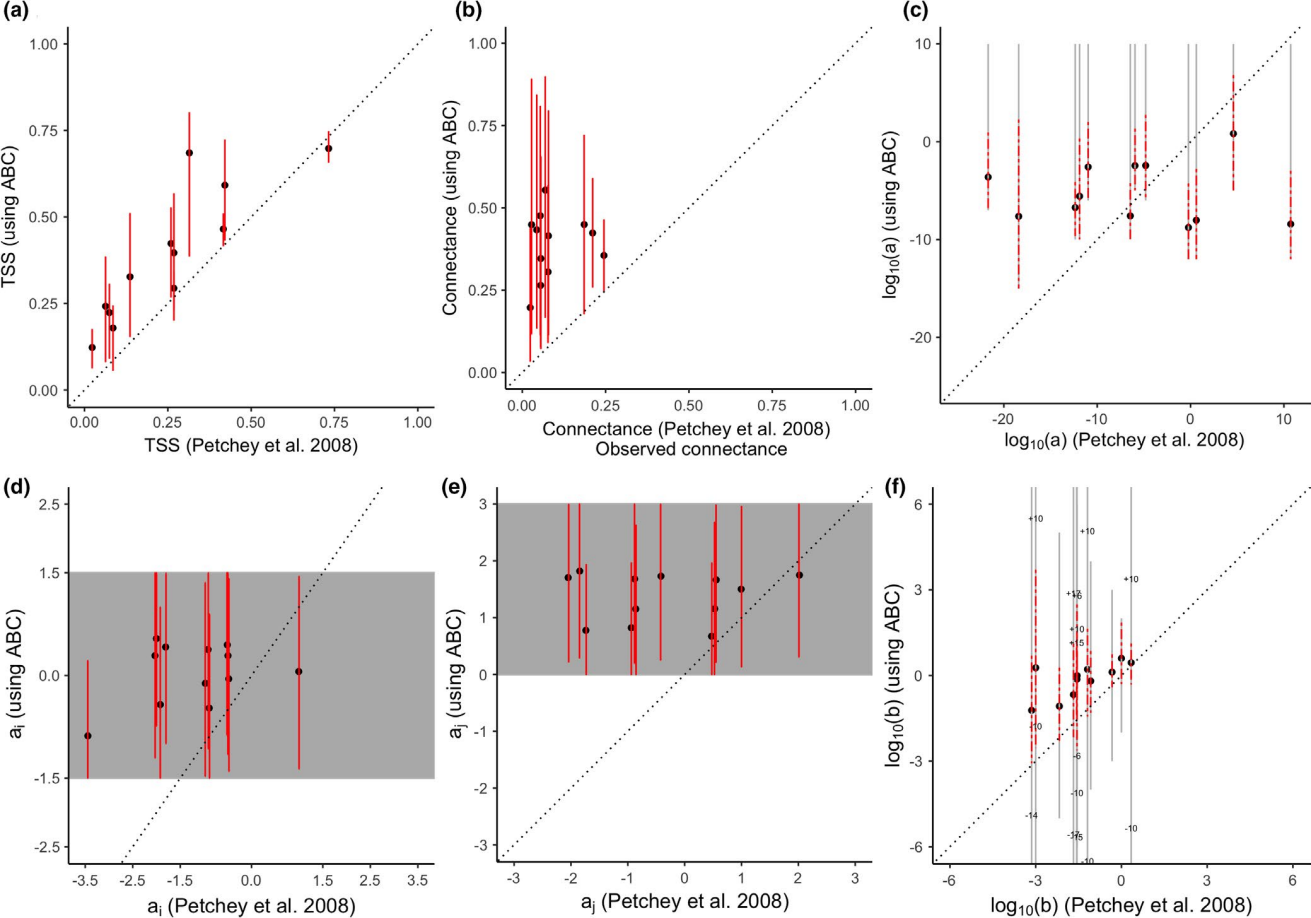
TSS (a), connectance (b) and ADBM parameters (c, d, e, f) computed using the ABC method compared with the corresponding point estimates from Petchey et al. (2008). The red lines are the 95% credible/prediction intervals and the black filled circles represent the corresponding means. The gray region represents the intervals of the prior distributions for $a_{i}$ and $a_{j}$. The gray lines represent the prior range of the parameters a and b in the $log_{10}$ scale. The prior range for the parameter b extends above and below the y‐axis limits for some food webs and so the values of the limits are shown on the plot. The dashed black lines are the 1:1 relationships for reference

The mean TSS using the ABC approach was higher than the point estimates from the PBRW study (Figure 5a) across all food webs except one. Our present approach led to estimates of connectance greater than the values of connectance of the PBRW study, which were fixed to equal the observed values of connectance.

We did not find a consistent relationship between the parameters estimated using the current approach and those estimated in the PBRW study (Figure 5c–f), except for in the case of parameter b. The mean using the ABC approach was always higher than the estimates from the PBRW study (Figure 5f) and the 95% credible interval of the posterior of b includes the estimate from the PBRW study.

The marginal posterior of parameter b was more constrained than the other three allometric parameters, that is, the posterior range was the narrowest (Figures S14–S25). In all of the food webs except Grasslands, the parameter b had a unimodal distribution (Figures S14–S25), whereas Grasslands had a bimodal distribution.

**FIGURE 6:**
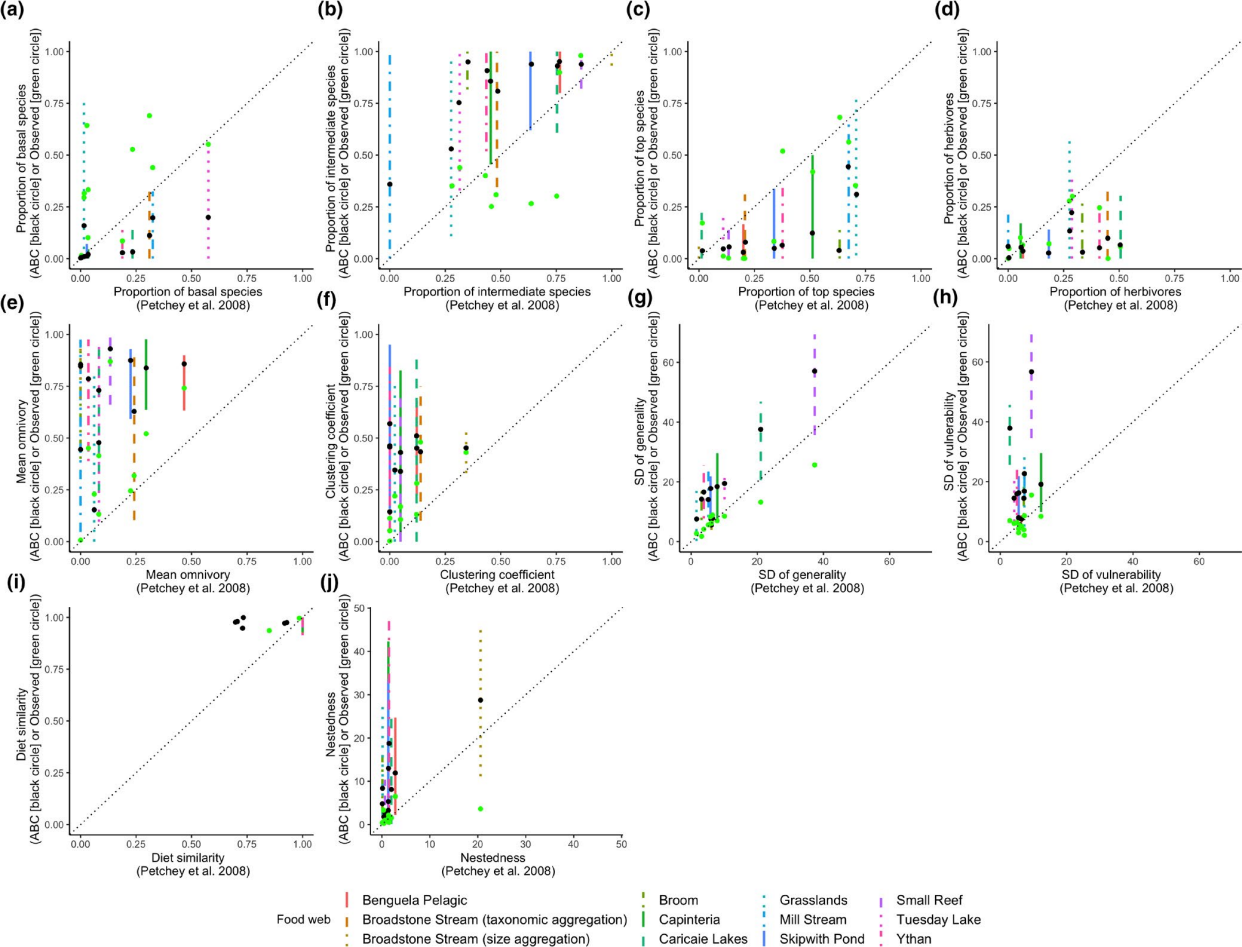
Structural properties of predicted food webs with 95% prediction interval parameterized using the ABC method plotted against the point estimates from Petchey et al. (2008). The black filled circles correspond to the mean, and green filled circles correspond to the properties of the observed food webs. The dashed black lines are the 1:1 relationships for reference

Compared to the estimates in the PBRW study, the proportion of intermediate species, mean omnivory, clustering coefficient, sd of generality, sd of vulnerability, diet similarity, and nestedness estimated from the current ABC approach were generally higher (Figure 6b,e–j). The proportion of basal species, proportion of top species, and proportion of herbivores were generally lower (Figure 6a,c,d).

The observed values of the proportion of intermediate species, mean omnivory, clustering coefficient, sd of generality, sd of vulnerability and nestedness were mostly within the lower range of the predicted 95% interval by the ABC method. The proportion of basal species, proportion of top species, and proportion of herbivores were underestimated in comparison to the observed values for most of the food webs.

**FIGURE 7:**
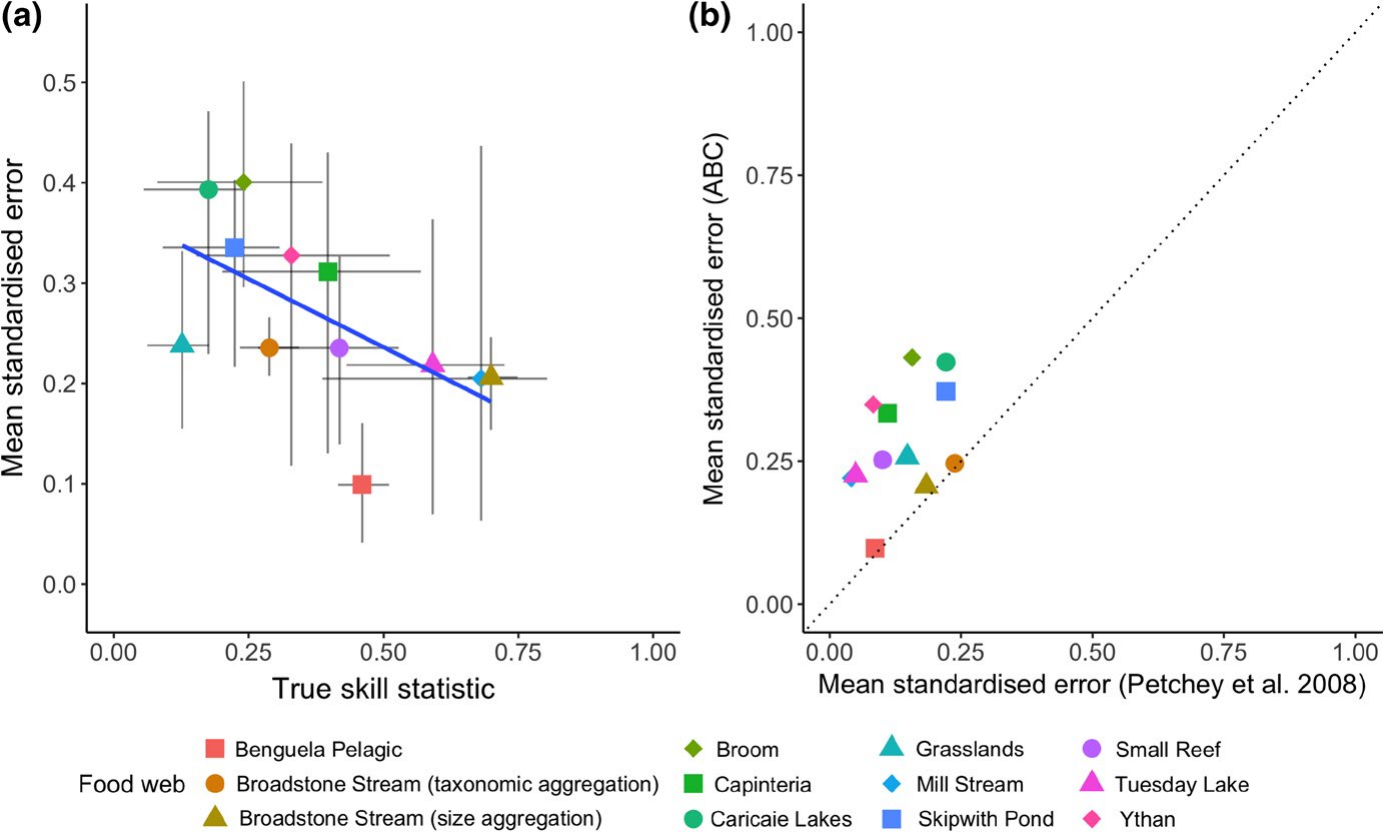
(a) The mean standardized error of the food web properties predicted from the ADBM parameterized using rejection ABC plotted against the mean TSS for each food webs. The vertical and horizontal bars correspond to 95% prediction intervals of the standardized error and TSS, respectively. Solid blue line is linear regression through the means (*t* = −2.335, *df* = 10, *p* = .041). (b) The mean standardized error computed from the ABC method plotted against the mean standardized error from Petchey et al. (2008). The dashed line is the 1:1 relationship for reference

The ADBM, when parameterized with the ABC, generally better predicted the structural food web properties, such as proportion of basal species when the TSS was higher (Figure 7a) across the 12 food webs. However, the ABC parameterized ADBM less accurately predicted food web properties on average than in the PBRW study (Figure 7b).

Within each food web, we found various relationships between the standardized error and TSS (Figures S30 and S31). For example, for Skipwith Pond food web (Figure S30h), high values of TSS were associated with high error, whereas the opposite was true for other food webs, such as Broadstone Stream (Figure S30b,l). The other food webs showed more complex relationships. The shapes of the point scatters in Figures S30 and S31 are caused by the structural constraints of the ADBM (e.g., it can only predict contiguous diets) interacting with the TSS and the connectance of the observed food web. These features prevent, for example, a predicted food web with very high connectance from having high TSS. Similarly, a predicted food web with connectance equal to that of the highest TSS prediction tends to have low TSS.

## 4 DISCUSSION

The ABC parameterization method employed here improves on the basic parameterization methods applied in Petchey et al. (2008) (PBRW). The ABC method provides uncertainty in parameter estimates and thereby a range of predicted food webs (Figure 5c–f). It also allowed us to estimate parameters that were fixed by the PBRW study and thereby also predicts connectance (Figure 5b). Including uncertainty and predicting connectance are significant advances because they allow predictions in changes of food web structure caused by environmental changes that include uncertainty in the predicted food web structure and including uncertainty in such predictions is critical (Cressie et al., 2009; Lindegren et al., 2010; Petchey et al., 2015). A future development will be to partition the contribution of different sources of uncertainty such as incomplete sampling and model deficiencies to make improvements in the model with the aim of reducing uncertainty. Future research should also investigate the functional and dynamical significance of the uncertainty in the predicted food web structure. Below, we discuss some of the results of our study and expand on these opportunities and priorities for future research.

### 4.1 Connectance and missing links

In all cases, the predicted connectance was greater than the observed connectance (Figure 5b). This result is similar to that of a recent study of European vertebrate food webs by Caron et al. (2022). They found that local food web connectance was consistently overestimated by a trait‐based model which used diet, nesting habitat, activity time, foraging behavior, and body mass of species.

Why was the predicted connectance greater than the observed connectance? Firstly, it is important to recognize that the ADBM (when using only body size as a trait) can only predict diets that are contiguous with respect to the size of prey. That is, it cannot predict that a predator will consume prey of sizes 1 and 3 and not prey of size 2. Such patterns can however be predicted if a trait in addition to size which is not perfectly correlated with size, influences foraging parameters (Allesina et al., 2008; Caron et al., 2022; Petchey et al., 2008; Stouffer et al., 2006). Secondly, it is important to note that the observed diets were not contiguous when prey are ordered by their size. This could be due to some ecological differences in how predator groups choose their prey (Caron et al., 2022). Hence, the estimation process will result in a greater number of predicted links than observed given these features, and the model attempts to maximize the coincidence of predicted and observed link presence and absence (i.e., the TSS).

These findings raise the question as to whether the model or the observed data are incorrect. We expect that some of the links that do in reality occur are not present in the empirical datasets. This could be caused by low empirical sampling effort or rare prey–predator interactions even when sampling is extensive. In this case, a false positive may actually be a correctly predicted link. More intensive and more complete sampling of links in food webs has been recognized as important, due to the potential that a low sampling effort will influence the perceived food web structure (Martinez et al., 1999).

We expect there are also cases of real false positives, where the model predicts a feeding link despite no possibility that such a link could occur in reality. This may be the case when a trait other than, or in addition to, prey size is influential. For example, a particular prey species may have a defensive trait that means it takes longer to consume it than an undefended prey of the same size. Incorporating traits other than body size in the ADBM would allow for discontiguous diets along the size axis. It is also possible that better estimates of parameters that could result from acquisition of new empirical data could cause lower estimated values of connectance. Furthermore, the ADBM's current form is a biology‐only model; it does not include an observation process, although this could be included. The model would then be able to predict the absence of a link due to incomplete observations.

It would be interesting to take a very well‐sampled food web (real or simulated) and remove links at random to create a less well‐sampled version, and to test if the very well‐sampled version can be predicted from the less well‐sampled version (with ABC parameter estimation). If it could, then there is potential to compensate for under‐sampling with an appropriate food web model and estimation procedure.

The ABC parameterization resulted in a lower prediction accuracy of structural features of the food webs (Figure 7b) due to the overestimation of connectance. This was confirmed by principal component analysis of variation in the food web structural properties which revealed a first PC axis representing on average 62% of the overall variance, and this first axis was highly correlated with connectance, with an average Spearman correlation of 0.87 (see SI S8 for details). Furthermore, there was a strong positive relationship between the mean standardized error in structural properties of the food webs and mean standardized error in connectance of the food webs (Figure S32).

### 4.2 Observing and predicting link absences

Our parameterization approach was to maximize the TSS (the coincidence of predicted and observed link presences, and the coincidence of predicted and observed link absences). The TSS assigns equal importance to the collection of presence and absence of observed links with the weight of an observed single presence or absence link being dependent on the connectance of the food web. If the connectance is <0.5, the TSS assigns more weight to a presence of link than to an absence of a link and vice versa.

Because the connectance of the observed food webs is <0.5 (Table 1), the TSS implicitly assigned more weight to a presence of link than to an absence of link. This upweighting of link presences seems appropriate since observing a feeding interaction is unambiguous, whereas not observing one may be caused by various processes. That is to say, the observation of a single feeding interaction is sufficient to record the presence of a link, whereas this is not true for the absence of links: One observation of a predator not consuming a prey does not mean that it will never do so.

To improve our estimation procedure, we could quantify the uncertainty in the recorded absence of links and include this uncertainty in the parameterization method. Weight/importance could be assigned to true positives, true negatives, false positives, and false negatives calculated from empirical studies which may be specific to that food web. Alternatively, an observation process could be added to the model, such that the biological part of the model can predict that a feeding link is possible, but then the observation process in the model leads to that link not being predicted.

### 4.3 Allometric parameters

In the PBRW study, the parameter b played a major role in maintaining the maximum predictive power of the ADBM. Indeed, they found that estimating b only, and not estimating either $a_{i}$ or $a_{j}$ only slightly decreased model performance, and that estimating only b and $a_{j}$ did not decrease model performance relative to when all three parameters were estimated.

We found that the posterior distribution of the parameter b was the most constrained of all the parameters (Figure 4). Parameter b defines the range of prey body size which has a finite handling time, and the prey size with the highest energetic profitability. As the parameter b relates to the prey–predator body size ratio, the constrained posterior of b (Figure 4d) recapitulates the importance of the ratio of body size of prey and predator in determining the food web structure with the ADBM.

The marginal posterior of parameter a was right‐skewed (Figure 4c). This may be because the ABC parameterization overestimates the connectance, which means that lower values of a are preferred over higher values of a (a lower value of a leads to a lower space clearance/attack rate, and a lower space clearance rate results in a higher connectance).

### 4.4 Incorporating other observed data

Information about who eats whom can be collected from multiple sources, such as gut contents of organisms, stable isotope composition of tissues, and experimentation (Layman et al., 2007; Peralta‐Maraver et al., 2017; Warren, 1989). Moreover, experimentation provides independent estimates of allometric foraging parameters, such as b, $a_{i}$, and $a_{j}$ (Rall et al., 2012). Diverse data could be used to parameterize the ADBM's predictions to test how uncertainty in the different datasets influences the ADBM's predictions using ABC. Appropriate summary statistics in the ABC method could be used to address such challenges. We could use, as an example, the approximate trophic position inferred from stable isotope ratio data from an individual tissue and gut content data of a predator simultaneously to parameterize the ADBM. The trophic position and the gut content information would be the summary statistics in this example. A further question that could be addressed in future studies is how the quantity of data affects the ADBM's predictions. The outcome of such a study could help food web researchers decide on how much data from a specific source is needed to predict the food web structure and help further optimize the deployment of limited sampling resources.

When only partial food web data are available (Patonai & Jord'an, 2017), the summary statistics in ABC can be used to infer these food web structures from the ADBM. It would be possible to use gut content data of only some of the species in a food web to parameterize the ADBM and predict the food web structure. Summary statistics opens up a broad spectrum of possibilities in parameterizing food web models. There are multiple empirical and theoretical studies on a range of different properties of food webs across different ecosystems (Goldwasser & Roughgarden, 1993; Martinez, 1991; Williams & Martinez, 2000). These can conceivably be used in parameterizing food web models using ABC to constrain the model predictions.

## CONFLICT OF INTEREST

None declared.

## AUTHOR CONTRIBUTIONS

**Anubhav Gupta:** Conceptualization (equal); Data curation (lead); Formal analysis (lead); Investigation (lead); Methodology (lead); Project administration (lead); Software (lead); Validation (lead); Visualization (lead); Writing—original draft (lead); Writing—review and editing (equal). **Reinhard Furrer:** Methodology (supporting). **Owen L. Petchey:** Conceptualization (equal); Funding acquisition (lead); Methodology (supporting); Resources (lead); Supervision (lead); Writing—original draft (supporting); Writing—review and editing (equal).

### OPEN RESEARCH BADGES

This article has been awarded Open Data and Open Materials badges. All materials and data are publicly accessible via the Open Science Framework at open Data: https://dx.doi.org/10.6084/m9.figshare.c.3298772, https://esapubs.org/archive/ecol/E092/066/#data, https://doi.org/10.5061/dryad.1mv20r6, https://zenodo.org/record/5575040 and Open Materials: https://doi.org/10.5281/zenodo.5735592, https://doi.org/10.5281/zenodo.5735584, https://doi.org/10.5281/zenodo.5735594.

## Supporting information

Supplementary Material

## Data Availability

All the data used in this study were collected in other studies and are openly available. We list those studies and the open‐access source in Table 1. The code used for data curation is available in the repository https://doi.org/10.5281/zenodo.5735592. The code used for fitting the ADBM using the point parameterization method in Petchey et al. (2008) is available in the repository https://doi.org/10.5281/zenodo.5735584. The complete code used in the analysis is available in the repository https://doi.org/10.5281/zenodo.5735594.
