# Supplementary material for: Simultaneously estimating food web connectance and structure with uncertainty
Source: Ecol Evol. 2022 Mar 8;12(3):e8643. doi: 10.1002/ece3.8643 (PMC8928887; doi:10.1002/ece3.8643)
Supplement: Supplementary file 1 — Supplementary Material [file ECE3-12-e8643-s001.pdf]

Supplementary Information:
Simultaneously estimating food web connectance and structure with uncertainty

Anubhav Gupta Reinhard Furrer Owen L. Petchey

13 February 2022

1 ABC methods

In the upcoming sections, we explain the Markov chain Monte Carlo and sequential Monte Carlo used to parameterise the ADBM.

1.1 Markov chain Monte Carlo ABC

Rejection ABC does not learn anything from rejection or acceptance events. It can therefore be inefficient, particularly when there is a high proportion of rejections. This may arise if prior distributions are wide and the observed data are informative as they may constrain the posterior distributions relative to the priors.

MCMC ABC attempts to overcome this shortcoming by learning from rejections and acceptances, and thereby focus tested sets of parameter values in regions of parameter space that have higher posterior probability, but still explore other parts of parameter space.

Based on Marjoram et al. (2003), the MCMC method is as follows:

Properties:

As in rejection ABC except with the addition:

- A proposal distribution $K(\theta|\theta')$ which is a normal distribution

Initialisation:

- Run the rejection method to choose a set of parameter values from $\theta_0 \sim \pi(\theta)$ within the distance tolerance

Sampling:

for $i = 1 \ldots n$

- Simulate $\theta' \sim K(\theta_i|\theta_{i-1})$
- Compute model result $x_i = model(\theta`)$
- Compute summary statistics $s(x')$
- Calculate $\alpha = \frac{K_h(d(s(x'),s(y)))\pi(\theta')K(\theta',\theta^{i-1})}{K_h(d(s(x^{i-1}),s(y)))\pi(\theta^{i-1})K(\theta^{i-1},\theta')}$
  - Simulate $u \sim U(0,1)$
  - If $u \leq \alpha$, $\theta^i = \theta'$ else, $\theta^i = \theta^{i-1}$

Output:

Construct posterior distribution using the correlated $\theta_1, \ldots, \theta_n$.

1.1.1 Burn in

There is a possibility that the posterior distribution is influenced by the initial set of parameter values θ_0. We therefore discard the first half of Markov chain and use only the second half.

1.1.2 Thinning

In the MCMC approach, the accepted parameter values are correlated as in some cases $\theta^i = \theta^{i-1}$; which reduces the independence of sampled parameter values in the posterior distribution. The Markov chains are thinned to reduce the dependence (autocorrelation < 0.05) which lowers number parameter values in the posterior distribution. The chain length was chosen such that the chain length was approximately 1000 after thinning.

1.1.3 Convergence

It is important to look into the convergence of the chains before drawing conclusions about the posterior distributions. The convergence was checked using Gelman-Rubin diagnostics. The value obtained from the Gelman-Rubin diagnostics should be less than 1.1 for convergence (Gelman and Rubin 1992).

1.2 Sequential Monte Carlo ABC

Sequential Monte Carlo ABC two main features: i. weighted resampling from the set of parameter values already drawn; ii. successive reduction in the distance threshold. This approach gives a set of posterior distribution where the successive posterior distribution is narrower than the previous one. SMC is more efficient than the rejection method when the posterior distribution is a lot narrower than the prior distribution. In SMC the parameters are uncorrelated and therefore do not require burn-in or assessment of convergence.

Properties:

As in rejection ABC except with the addition:

- A proposal distribution $K(\theta|\theta')$ which is a normal distribution
- A decreasing sequence of tolerance thresholds $\epsilon_1, \ldots, \epsilon_T$

Initialisation:

At iteration $t = 1$,

- for $i = 1, \ldots, N$,
  - until distance $d(S(x), S(y)) < \epsilon_1$
    * simulate model parameter $\theta_i^{(1)} \sim \pi(\theta)$ and model output $x = model(\theta_i^{(1)})$.
- Set equal probabilities $w_i^1 = 1/N$ for parameter values.
- Take variance of proposal distribution τ_2^2 as twice the empirical variance of the $\theta_1^{(1)}$'s.

Sampling:

At iteration $2 \leq t \leq T$,

- for $i = 1, \ldots, N$,
  - until distance $d(S(x), S(y)) < \epsilon_t$
    * pick θ_i^* from the $\theta_j^{(t-1)}$'s with probabilities $w_j^{(t-1)}$
    * generate model parameter $\theta_i^{(t)} \sim K(\theta|\theta_i^*; \tau_t^2)$ and model output $x = model(\theta_i^{(t)})$.
  - Set

$$w_i^{(t)} \propto \frac{\pi(\theta_i^{(t)})}{\sum_{j=1}^{N} w_j^{t-1} K(\theta_i^{(t)}|\theta_j^{(t-1)}; \tau_t^2)}$$

  - Take τ_{t+1}^2 as twice the weighted empirical variance of the $\theta_i^{(t)}$'s

Output:

Construct a sequence of converging posterior distributions using the parameter values: $\theta_1^t, \ldots, \theta_n^t$ for all $1 \le t \le T$

2 Choice of threshold value of distance

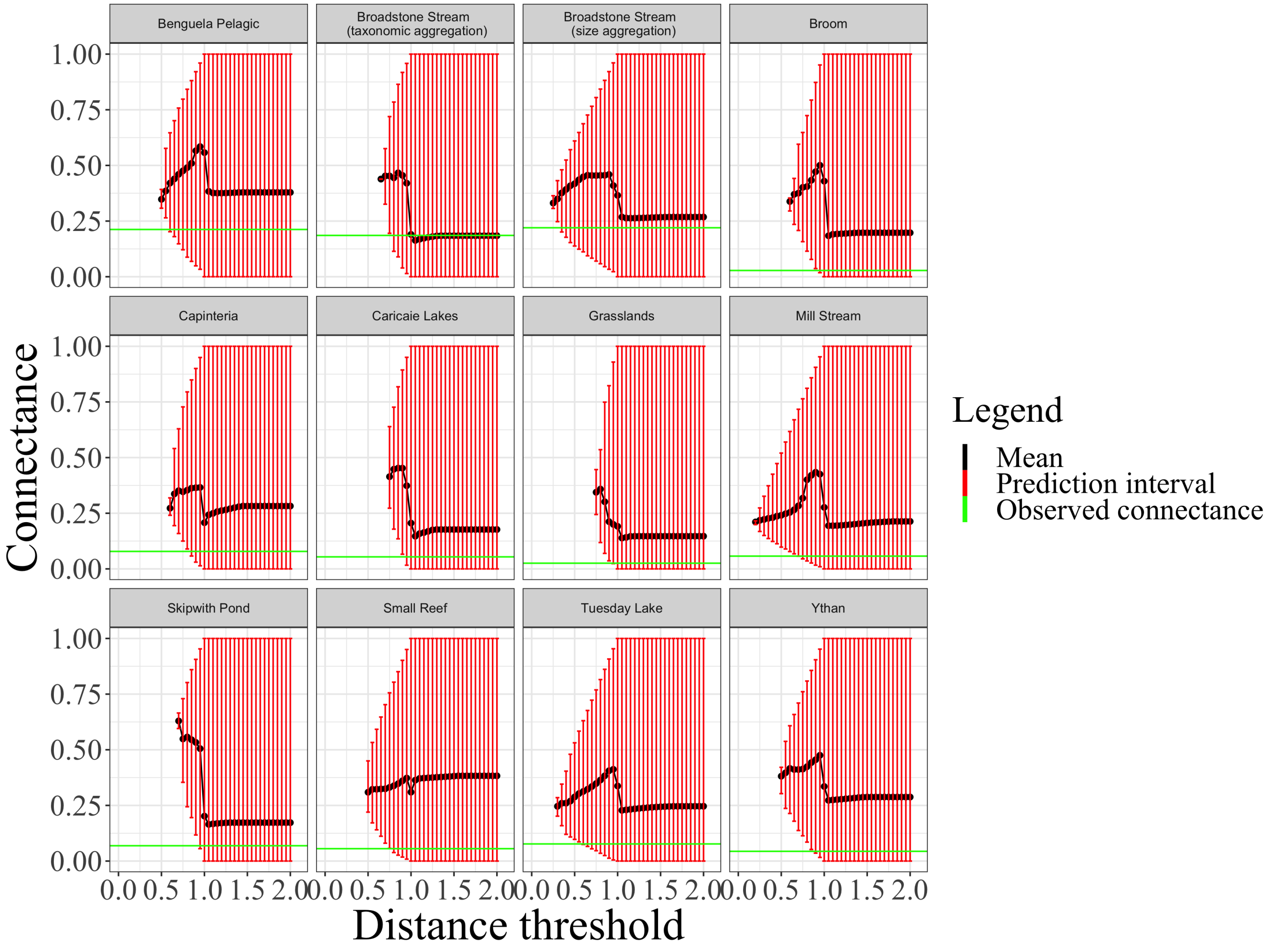

Figure S1: The prediction interval of the predicted connectance increases with increasing distance threshold. The green line and black line represent the observed connectance and mean of predicted connectance respectively.

3 Figure legends

Figure S2-S13: (a) Observed and predicted predation matrices of the given food web. Body size increases from left to right and top to bottom along the predation matrix. Black circles show where there were observed trophic links. The intensity of the pink circles shows the proportion of 1000 predicted food webs that had a trophic link between the corresponding species. This type of overlay is shown for two example predicted in panel (c). (b) The histogram of the number of times a link was predicted across the 1000 independently predicted food webs. The red bar shows the number of pairs of species for which a trophic link was never predicted. (c) Two predicted predation matrices correspond to the minimum and the maximum value of estimated b, and their sum.

Figure S14-S25: Marginal prior and marginal posterior distribution of the ADBM parameters for 12 food webs estimated using rejection ABC. The black vertical line in (d) corresponds to the value of b (=2) above which the most profitable prey item is larger in respect to the predator size.

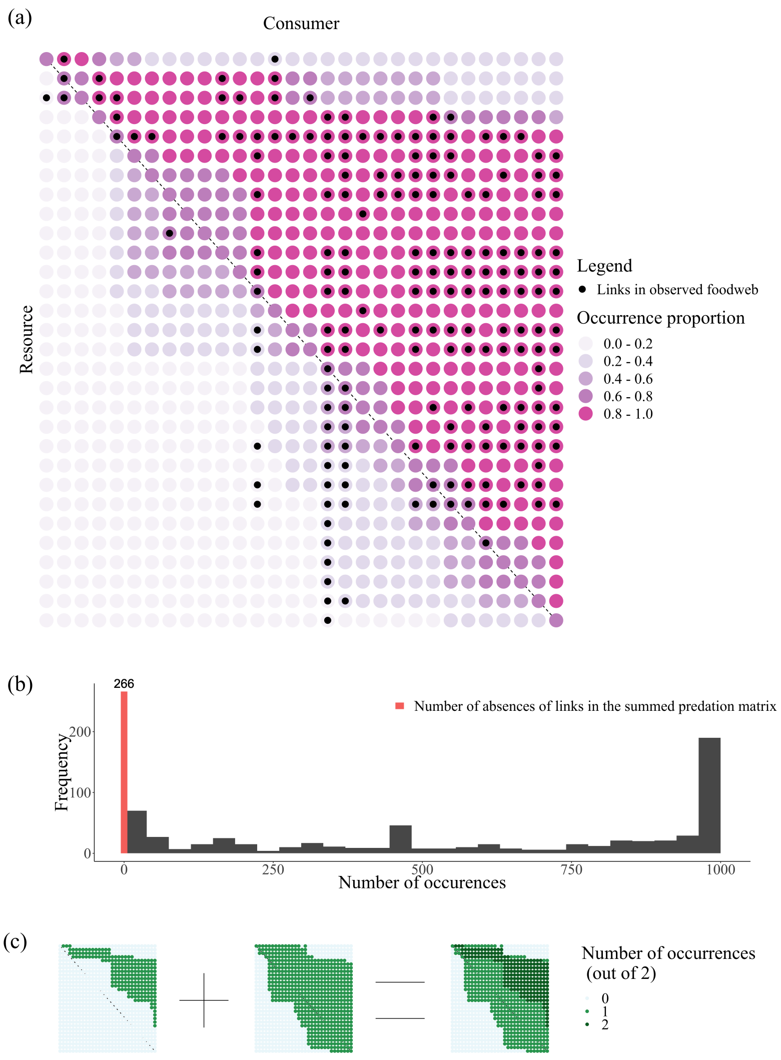

Figure S2: Benguela Pelagic

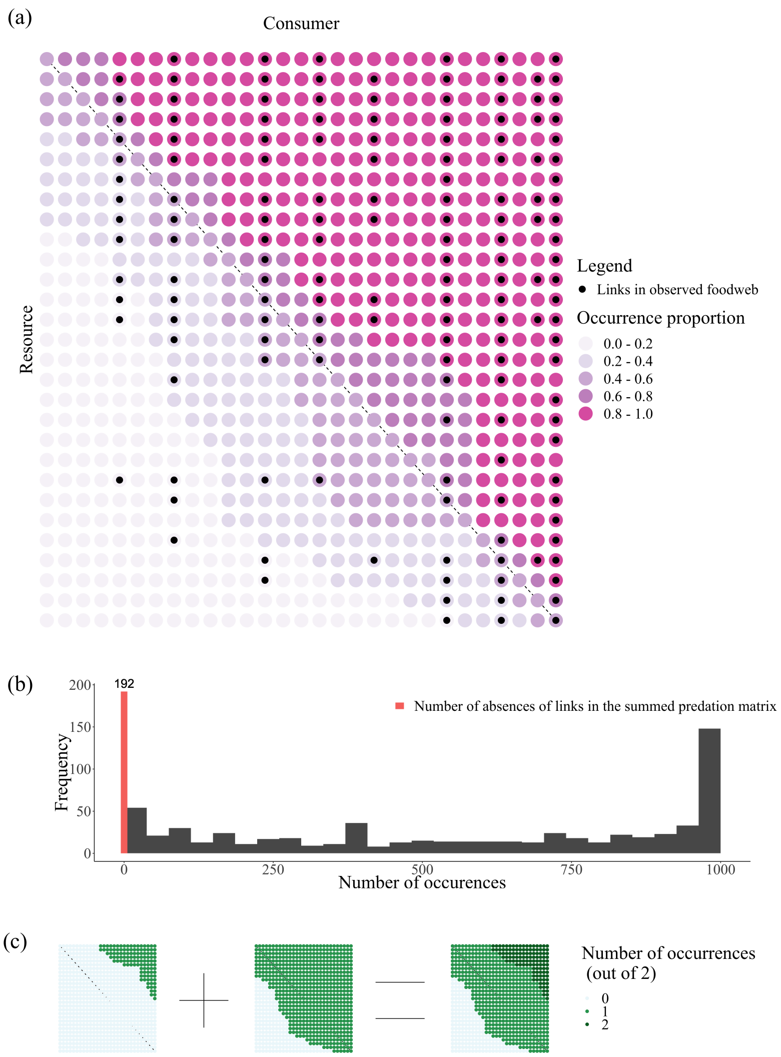

Figure S3: Broadstone Stream (taxonomic aggregation)

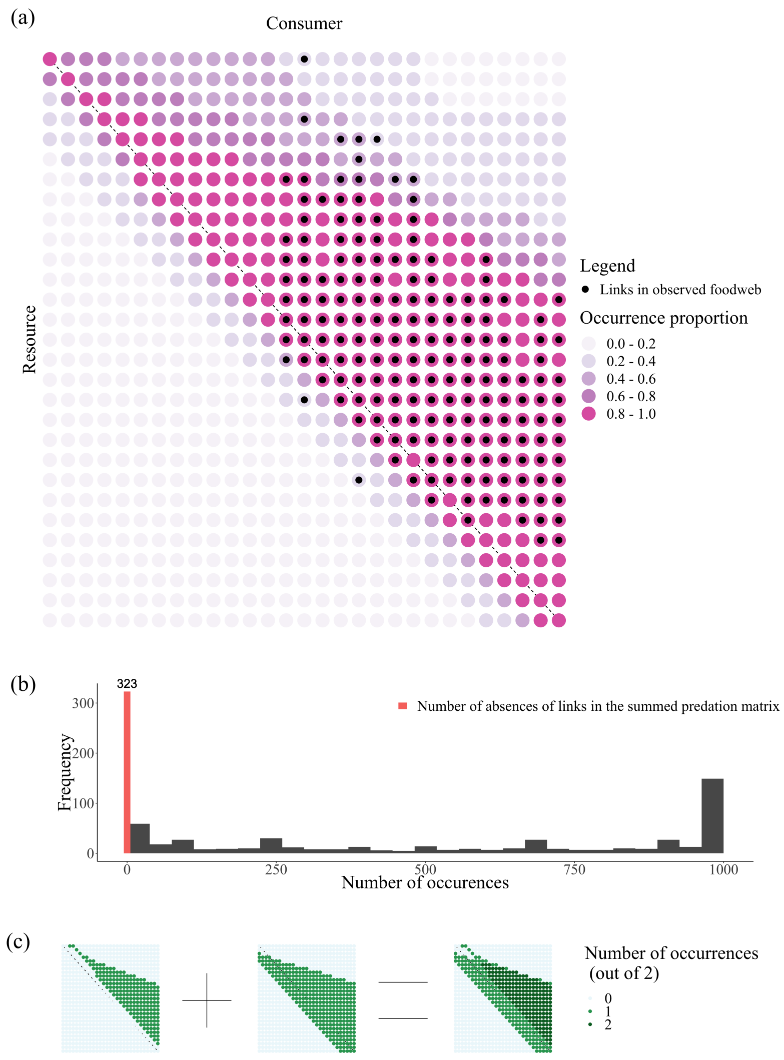

Figure S4: Broadstone Stream (size aggregation)

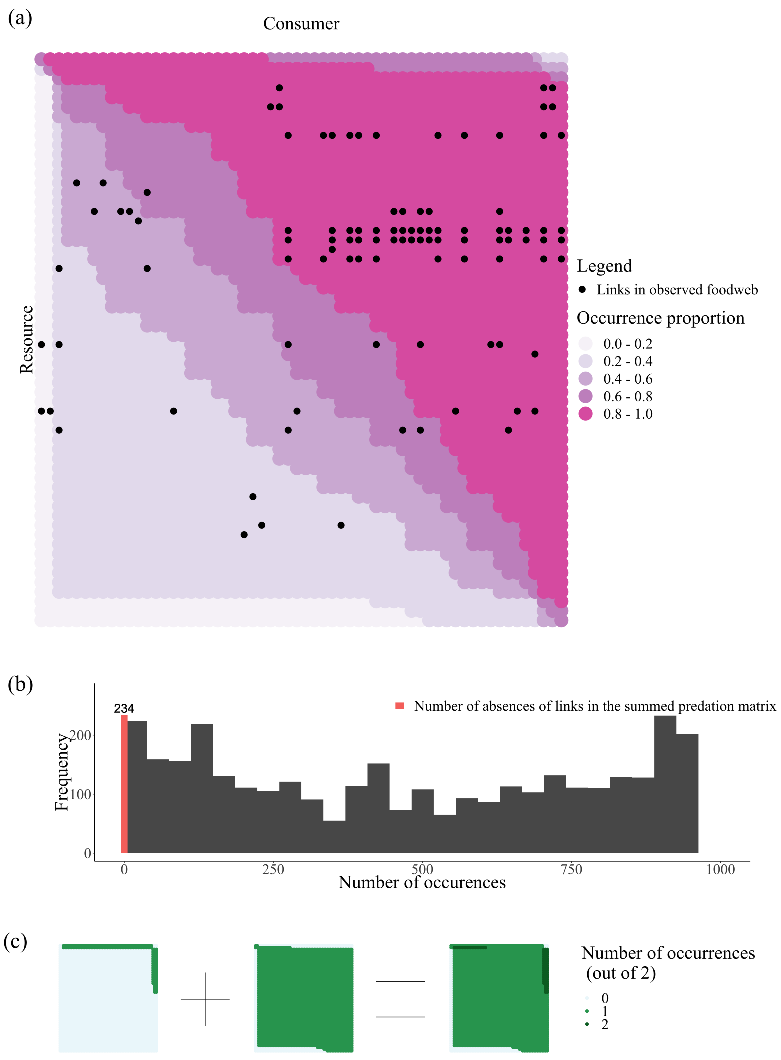

Figure S5: Broom

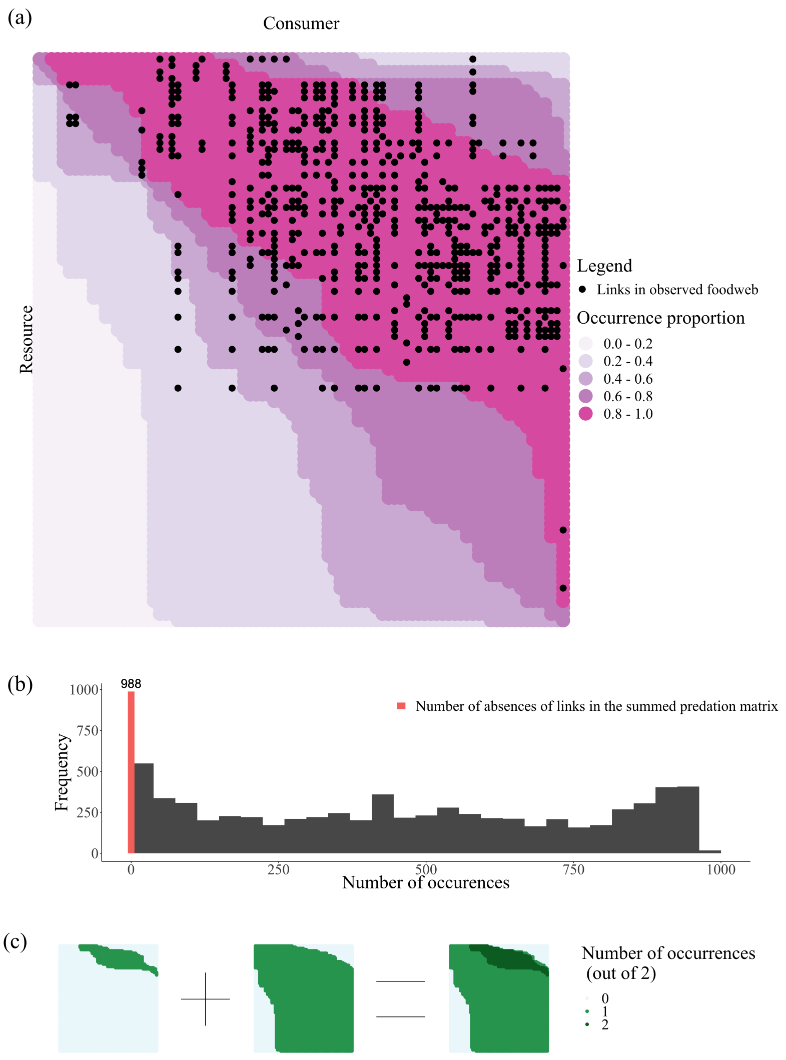

Figure S6: Capinteria

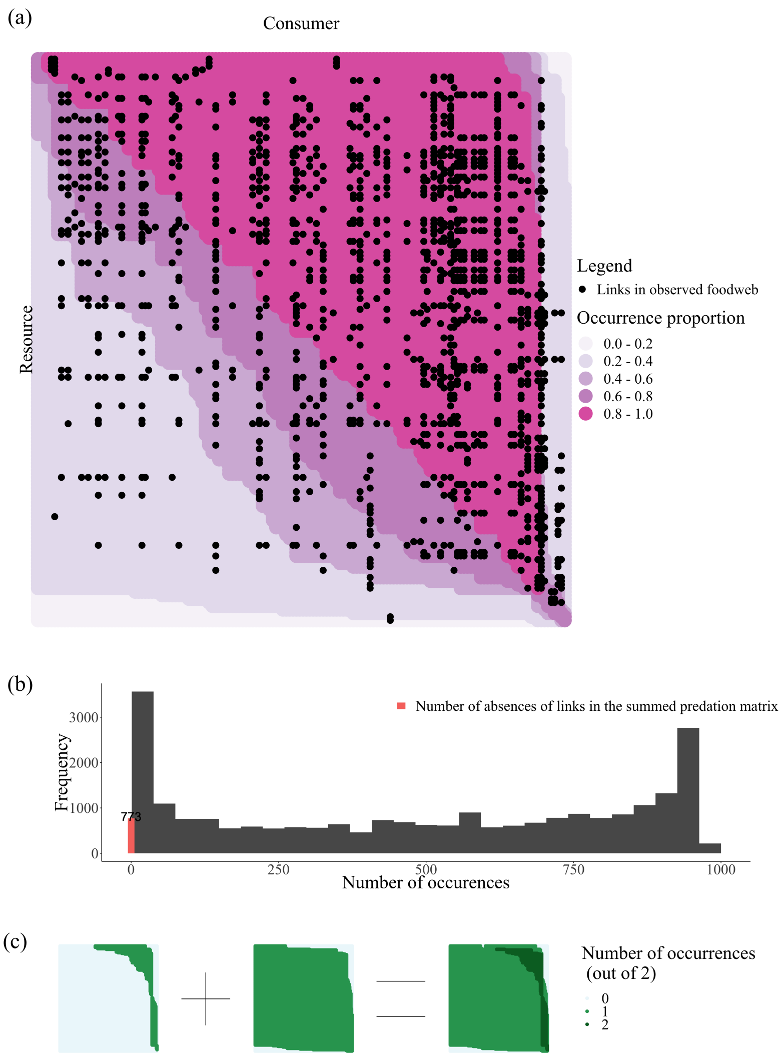

Figure S7: Caricaie Lakes

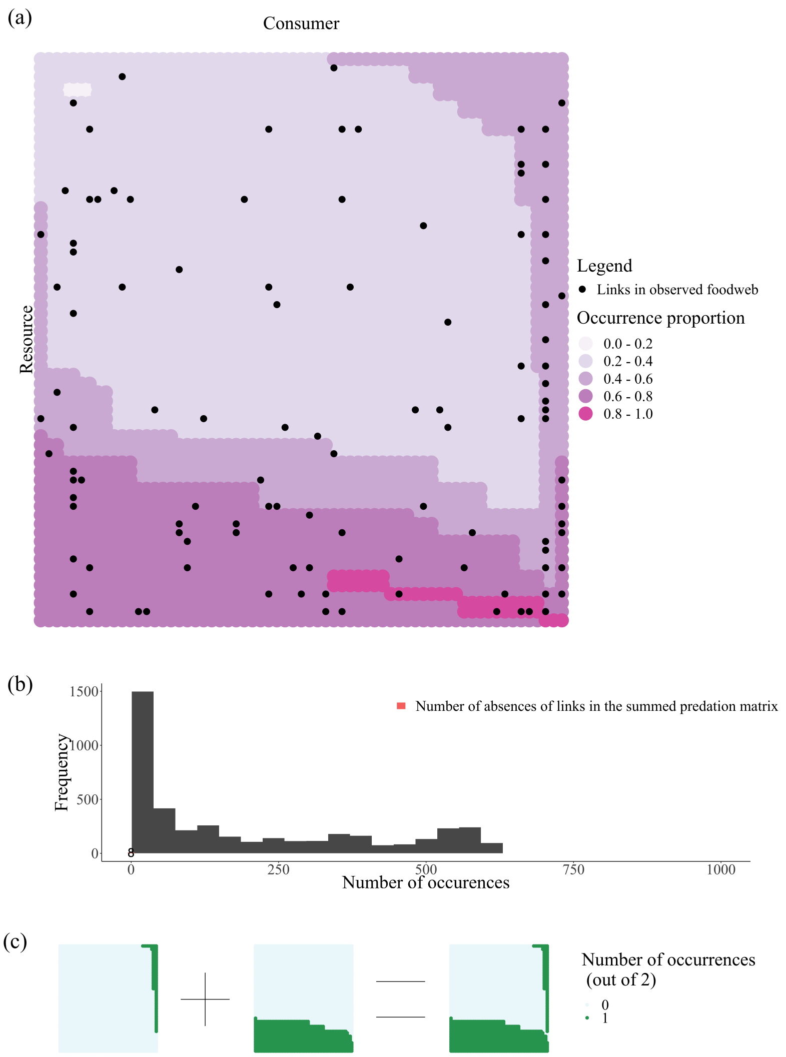

Figure S8: Grasslands

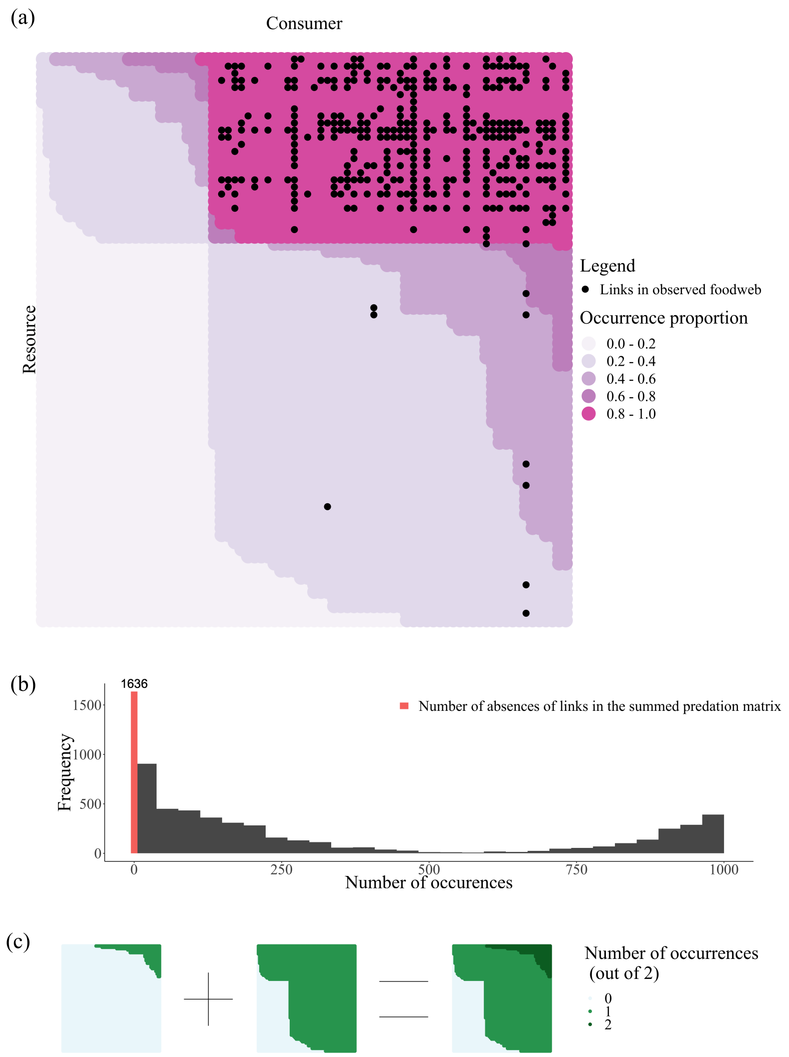

Figure S9: Mill Stream

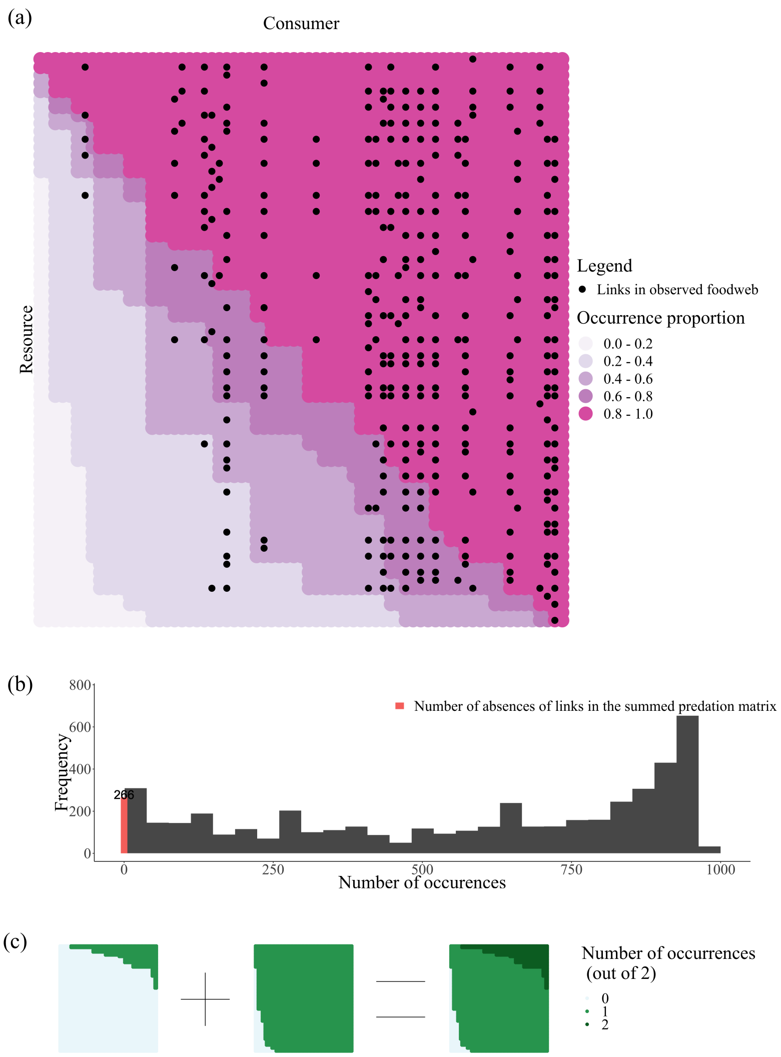

Figure S10: Skipwith Pond

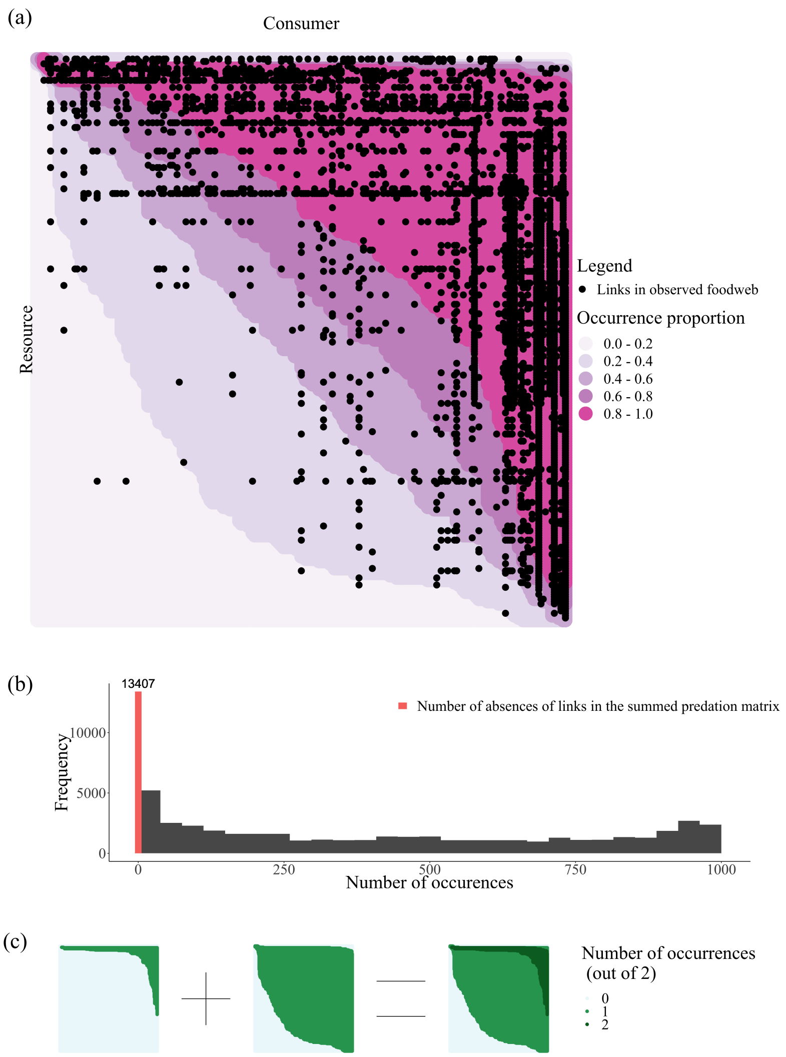

Figure S11: Small Reef

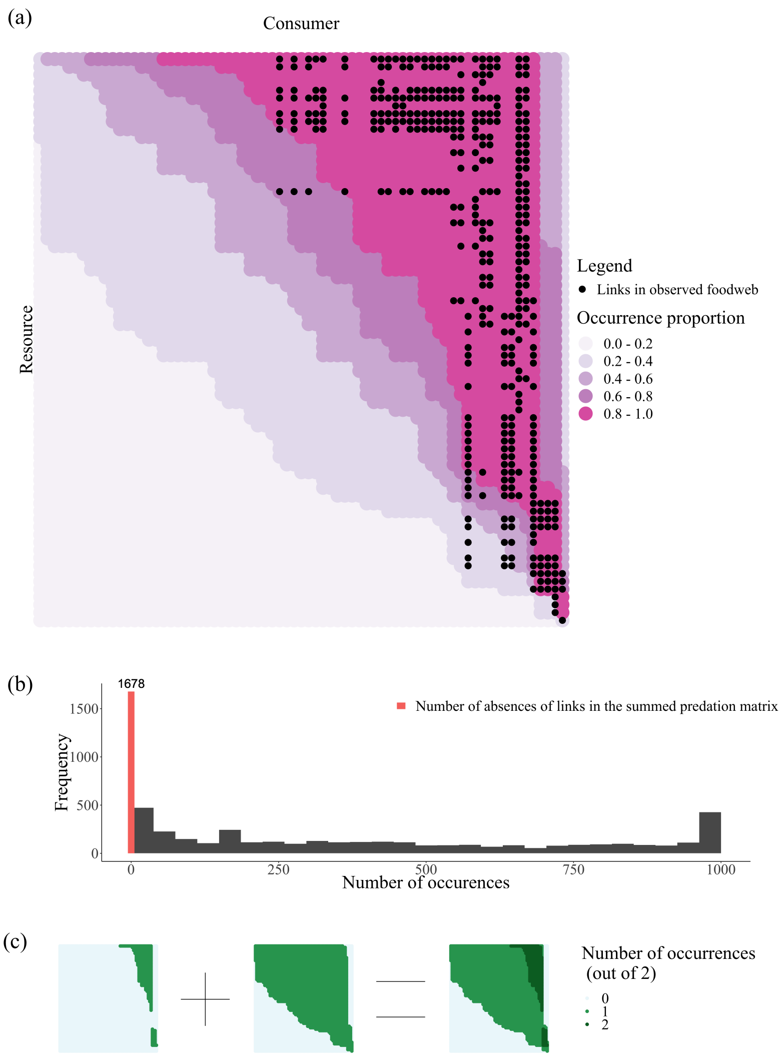

Figure S12: Tuesday Lake

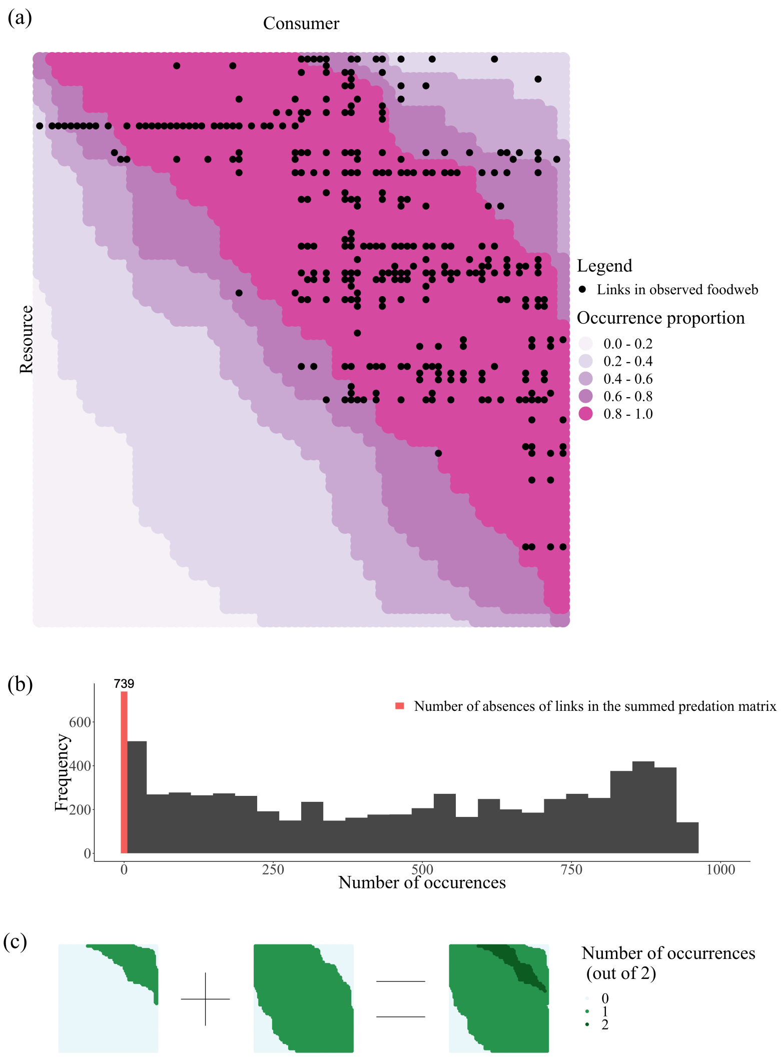

Figure S13: Ythan

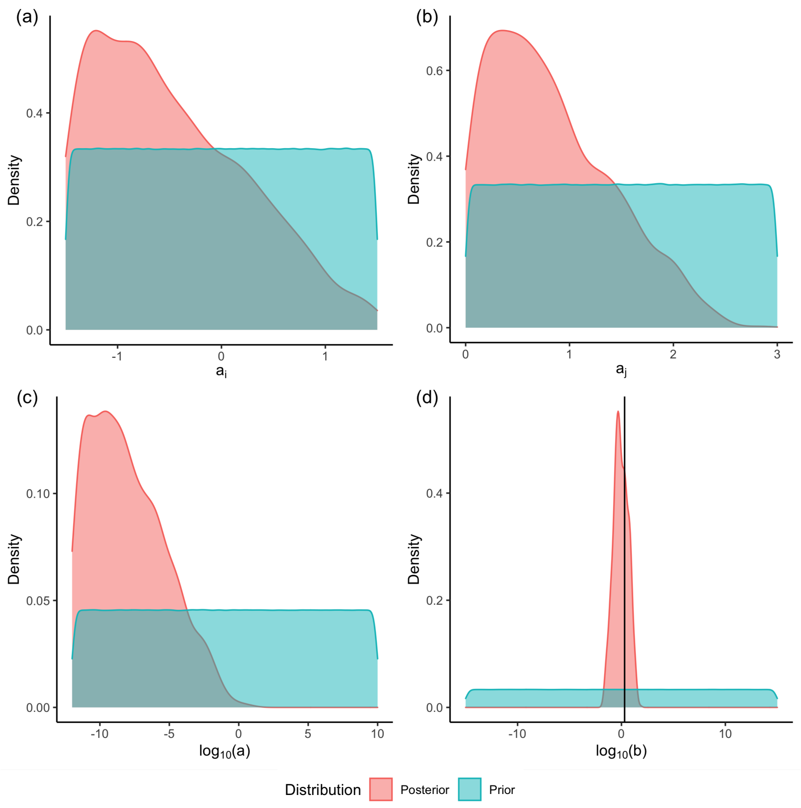

Figure S14: Benguela Pelagic

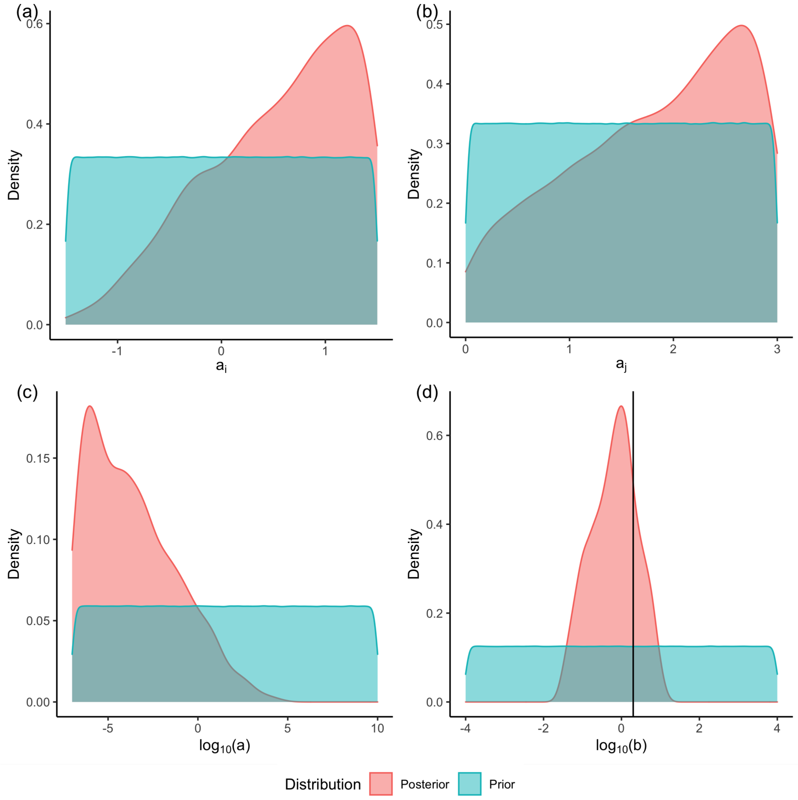

Figure S15: Broadstone Stream (taxonomic aggregation)

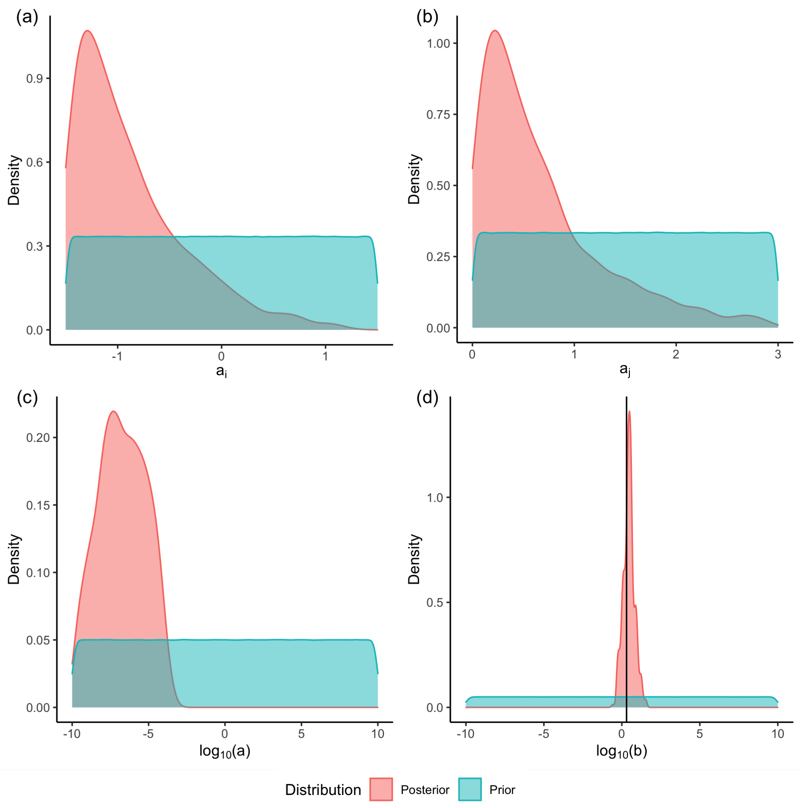

Figure S16: Broadstone Stream (size aggregation)

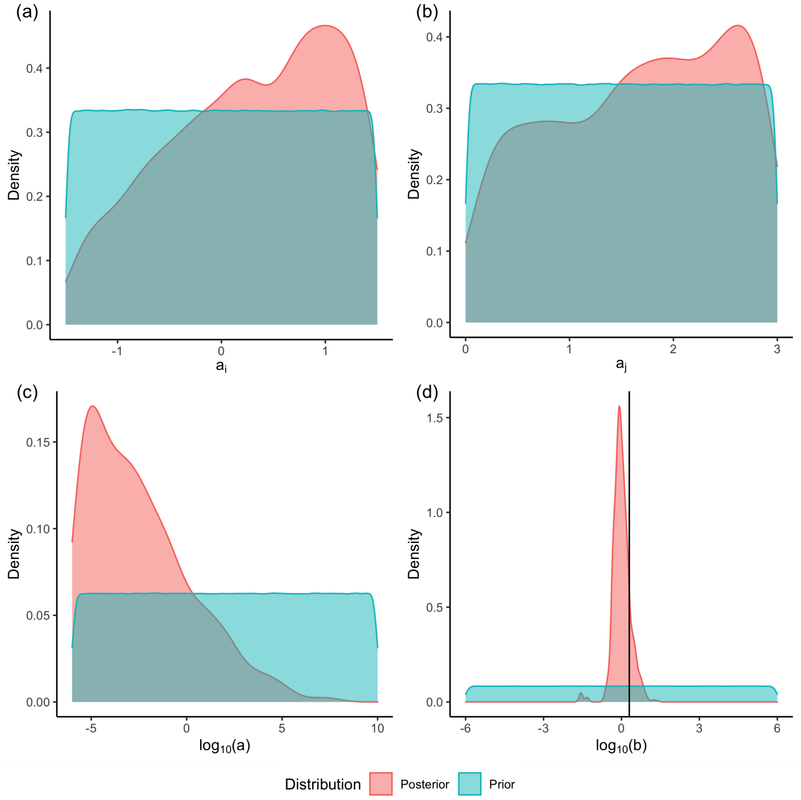

Figure S17: Broom

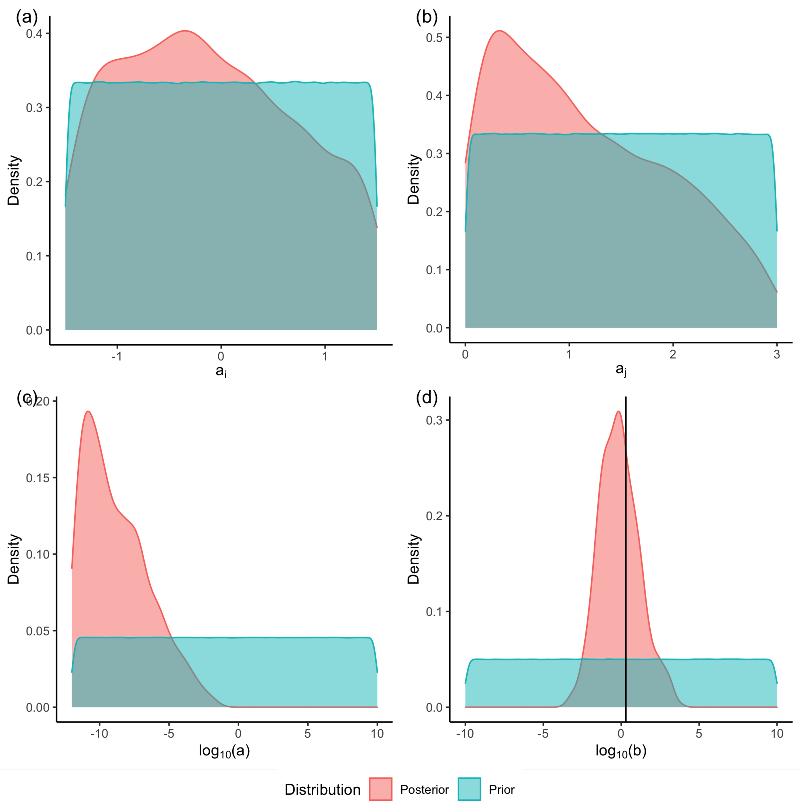

Figure S18: Capinteria

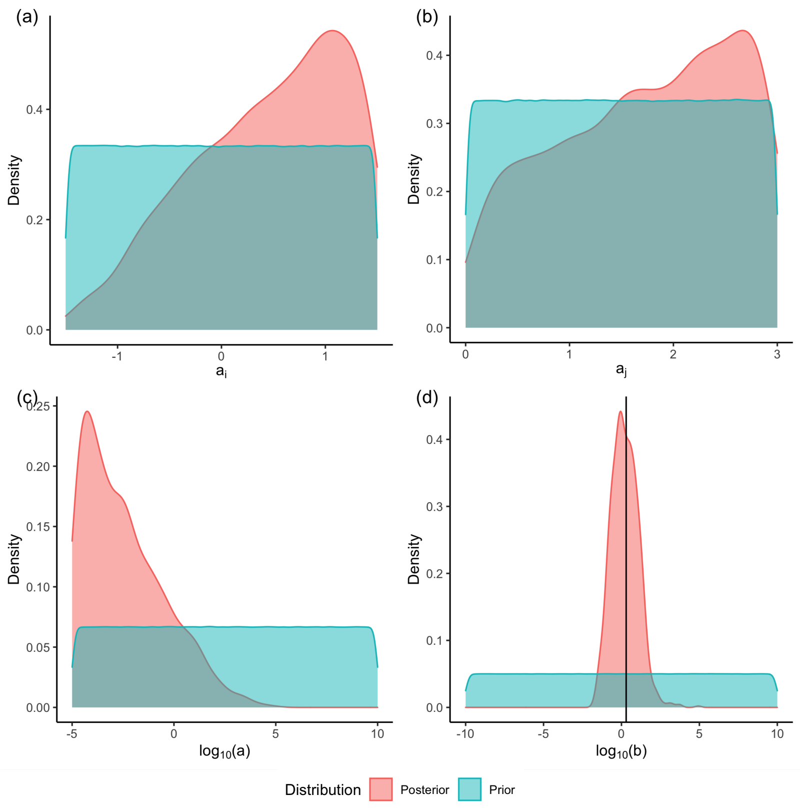

Figure S19: Caricaie Lakes

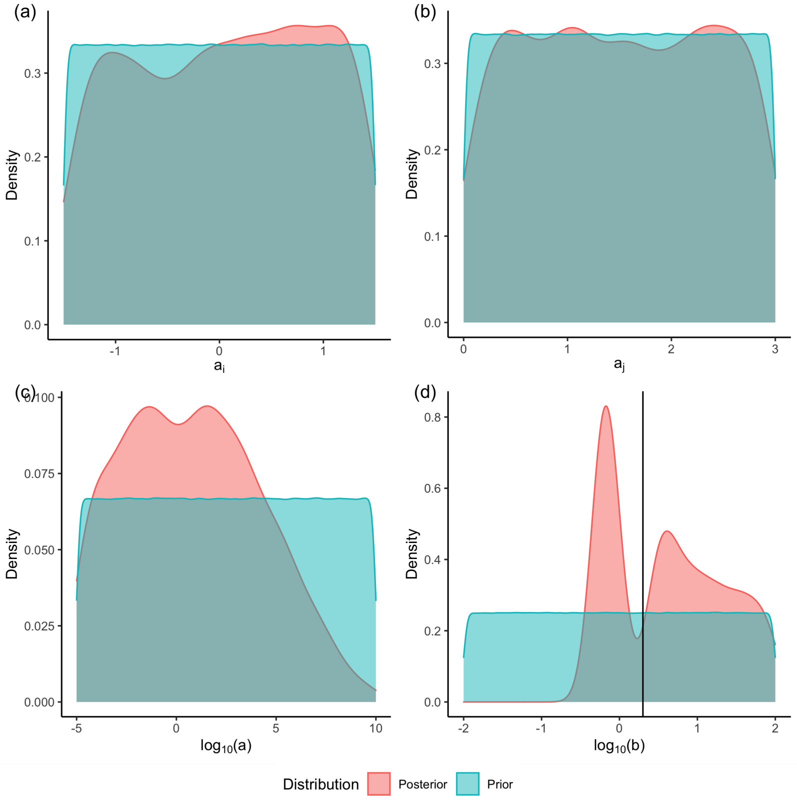

Figure S20: Grasslands

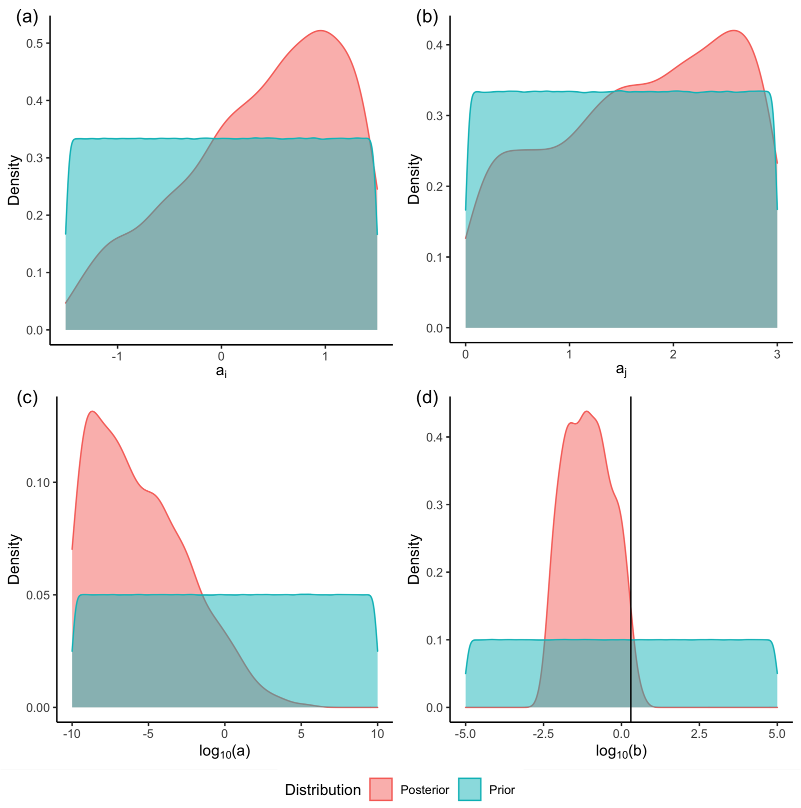

Figure S21: Mill Stream

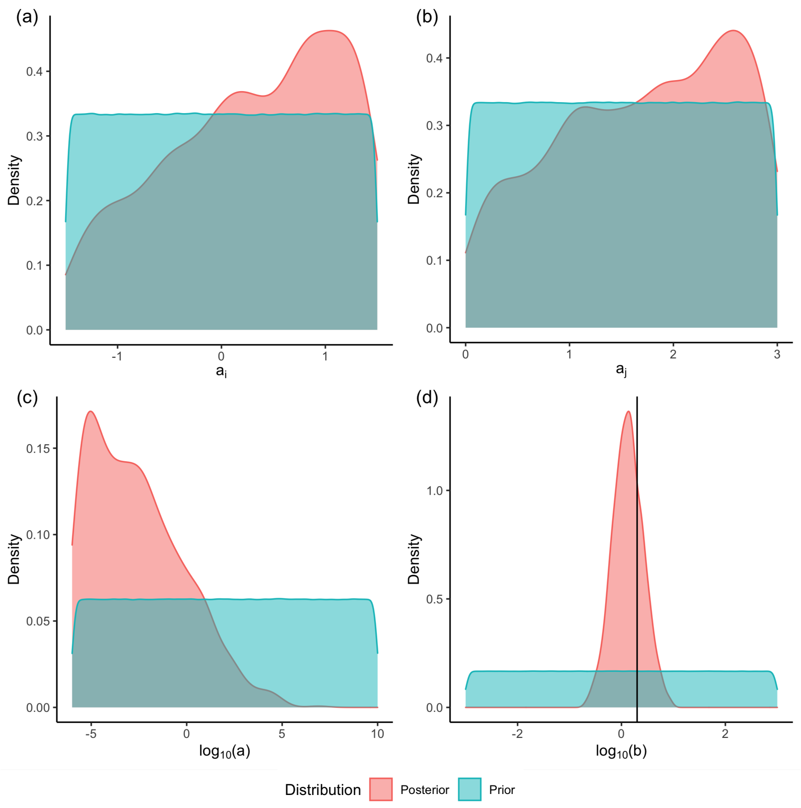

Figure S22: Skipwith Pond

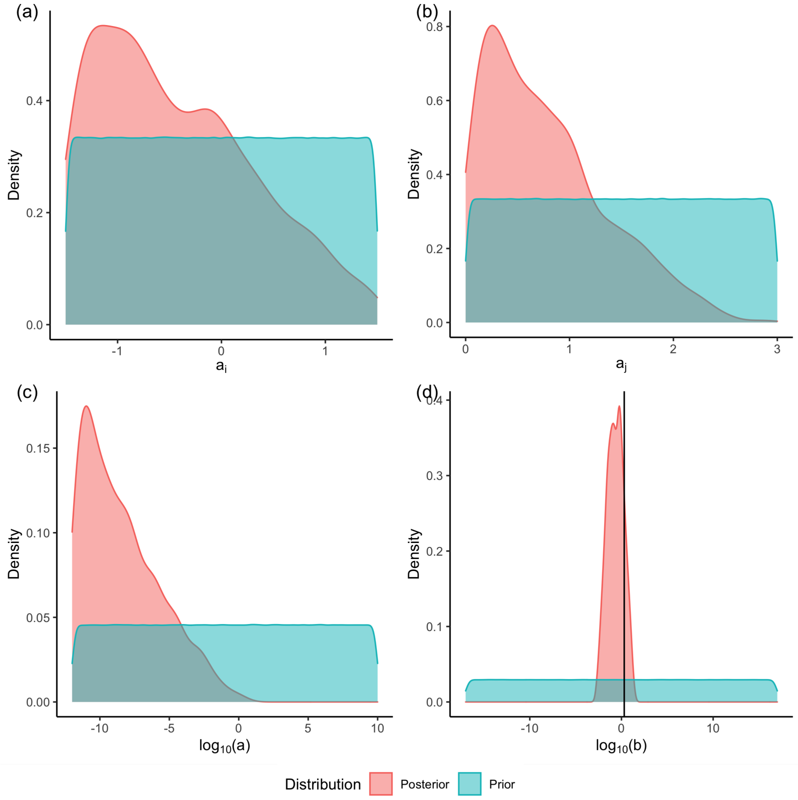

Figure S23: Small Reef

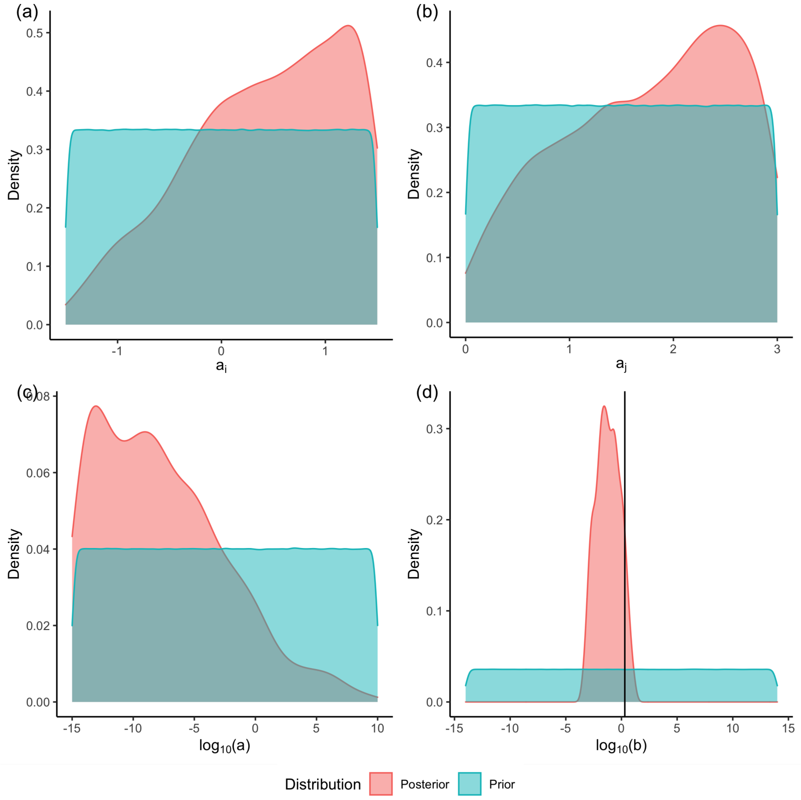

Figure S24: Tuesday Lake

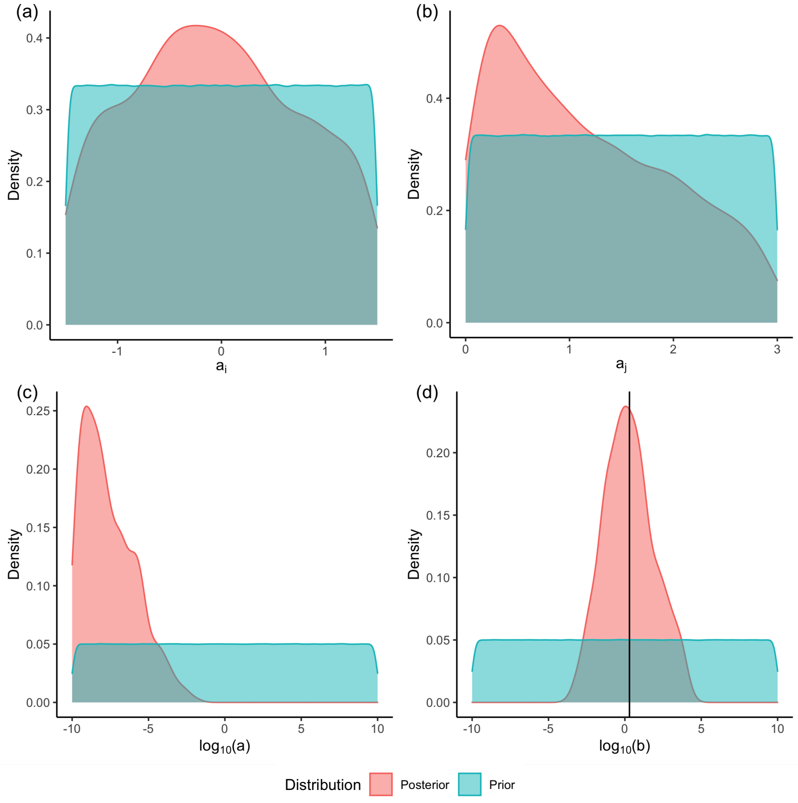

Figure S25: Ythan

4 Comparison between methods

We found consistent results among all three ABC methods (Fig. S27). The marginal posteriors of the ADBM parameters estimated using the three ABC methods for the Benguela Pelagic food web were quite similar in Fig. S26. In terms of simplicity, rejection ABC was the easiest to implement and resulted in similar results as the MCMC ABC and SMC ABC. To achieve similar number of accepted parameter values, MCMC ABC required a higher number of simulations, as MCMC ABC resulted in correlated parameter values and needed to be thinned. MCMC ABC was the most time consuming method. The SMC ABC method helped us to understand how the parameter distribution converged in response to the decrease in tolerance.

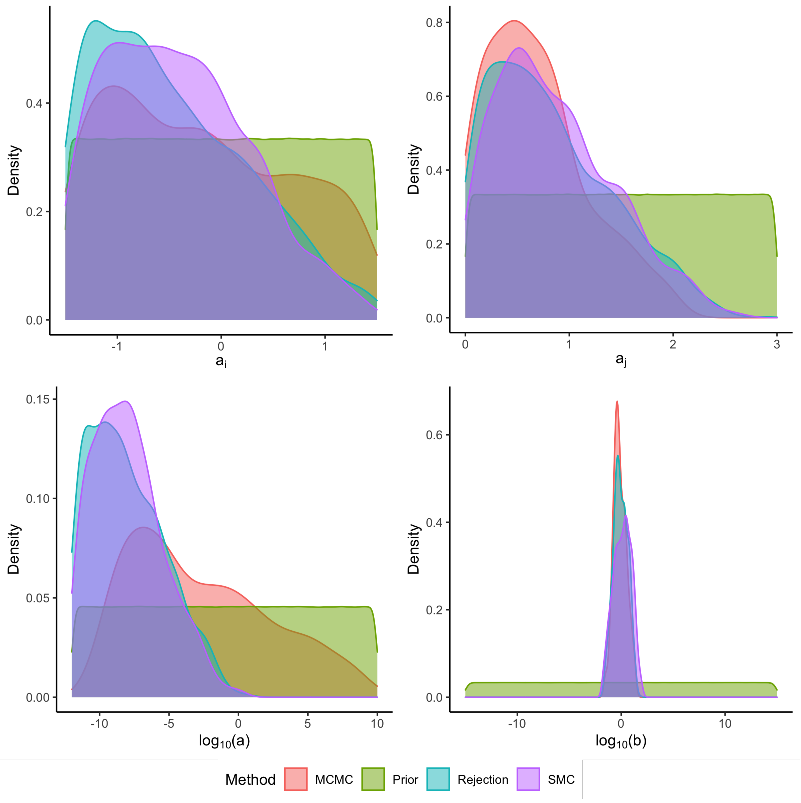

Figure S26: Marginal posterior distributions of the ADBM parameters estimated from rejection ABC, MCMC ABC and SMC ABC for Benguela Pelagic food web.

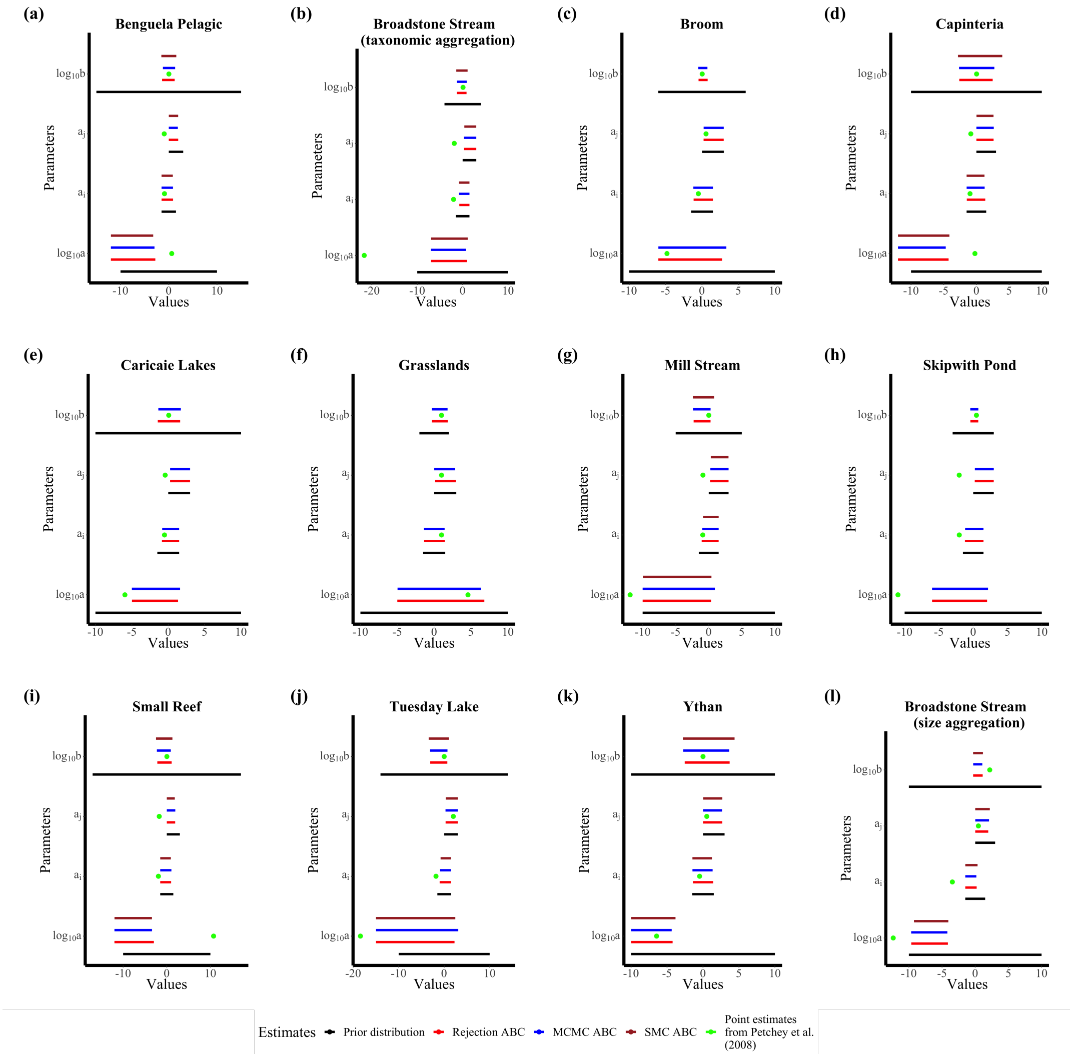

Figure S27: The 95% credible intervals of marginal posterior distributions of the ADBM parameters estimated from rejection ABC, MCMC ABC and SMC ABC for 12 food webs.

5 Model parameters

In the ADBM, the contingency model of optimal foraging predicts the diet k of each consumer j that maximises rate of energy intake given below:

$$\frac{\sum_{i=1}^{k} \lambda_{ij} E_i}{1 + \sum_{i=1}^{k} \lambda_{ij} H_{ij}}$$

$$= \frac{\sum_{i=1}^{k} A_{ij} N_i E_i}{1 + \sum_{i=1}^{k} A_{ij} N_i H_{ij}}$$

$$= \frac{\sum_{i=1}^{k} a M_i^{a_i} M_j^{a_j} n M_i^{n_i} e M_i}{1 + \sum_{i=1}^{k} a M_i^{a_i} M_j^{a_j} n M_i^{n_i} \frac{h}{b - \frac{M_i}{M_j}}}$$

$$= \frac{\sum_{i=1}^{k} nae M_i^{a_i+n_i+1} M_j^{a_j}}{1 + \sum_{i=1}^{k} nah M_i^{a_i+n_i} M_j^{a_j} \frac{1}{b - \frac{M_i}{M_j}}}$$

Since, the maximum of the above expression is affected by the product nah, estimating all the three parameters would be redundant, which would result in strong correlation. Therefore, it is sufficient to estimate any single parameter from nah, so we estimated only a. We set $n = 1$ and $h = 1$. Also, parameter e does not affect the value of k at which the above expression has a maximum, so value of e can be arbitrary.

6 Prior selection of a

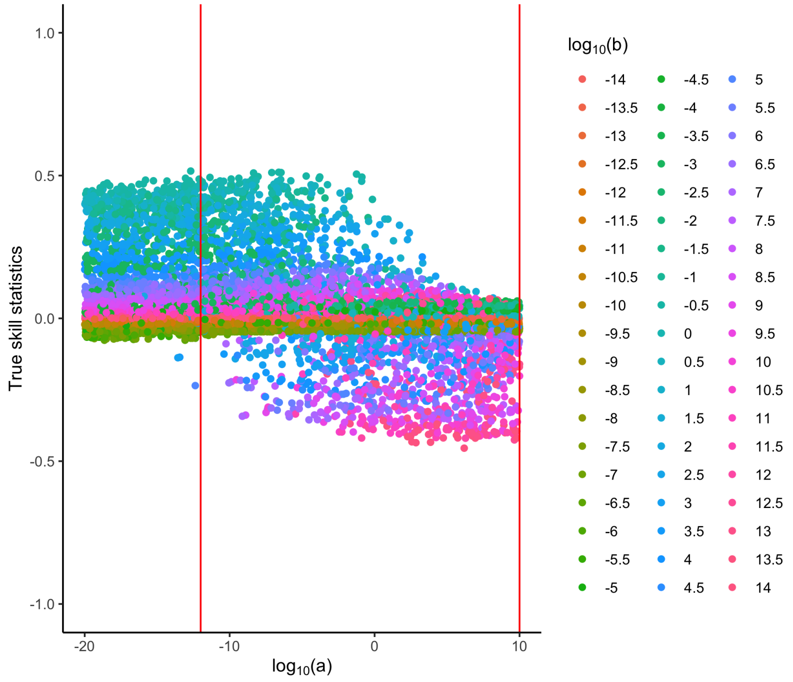

Figure S28: True skill statistics between the observed and predicted food web for Benguela Pelagic food web plotted against the attack rate scaling parameter a which is in log scale. The red lines represent the boundaries for the prior range of $log_{10}(a)$.

7 Parameter correlations

A few of the parameters were strongly correlated (Fig. S29). E.g., 0.71 correlation coefficients between a and a_i in Broadstone stream (size aggregation) food web. However, 98% of parameter pairs showed less than 0.5 correlation coefficient and 82% were less than 0.25. The highest correlation, 0.71 was due to somewhat redundant effects of the two parameters (a and a_i) on connectance, which is a mathematical feature of the model rather than anything of great biological significance.

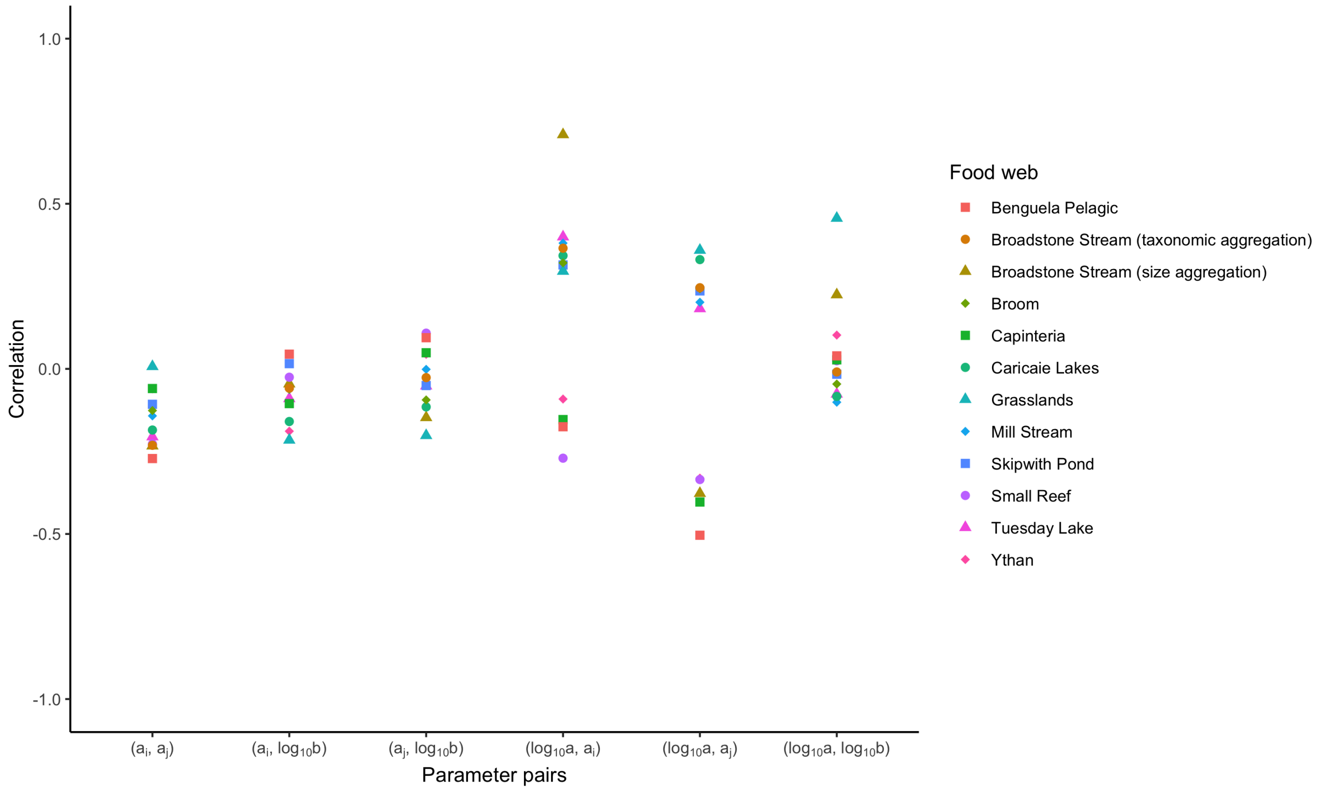

Figure S29: Correlations between parameter pairs of ADBM for 12 food webs.

8 Principal Component Analysis on structural properties

Table 1: Variance in structural properties explained by the first three principal compenents and correlation between connectance and first principal component.

Food web	Principal Component I	Principal Component II	Principal Component III	Correlation(PCI, Connectance)
Benguela Pelagic	0.66	0.20	0.07	-0.96
Broadstone Stream (taxonomic aggregation)	0.73	0.12	0.10	0.94
Broom	0.54	0.19	0.11	-0.89
Capinteria	0.65	0.13	0.10	0.92
Caricaie Lakes	0.59	0.18	0.10	0.85
Grasslands	0.57	0.20	0.10	0.74
Mill Stream	0.59	0.18	0.12	0.94
Skipwith Pond	0.61	0.19	0.11	0.89
Small Reef	0.67	0.11	0.09	0.95
Tuesday Lake	0.70	0.12	0.08	0.97
Ythan	0.64	0.18	0.08	0.86
Broadstone Stream (size aggregation)	0.47	0.25	0.17	0.60
Average	0.62	0.17	0.10	0.88

9 Summary table of linear regression between average standardized error and true skill statistic from Figs S30 and S31

9.1 Benguela Pelagic

```
##
## Call:
## lm(formula = mean_err ~ TSS, data = mean_err_TSS)
##
## Residuals:
##      Min       1Q   Median       3Q      Max
## -0.07694 -0.02674 -0.01177  0.03389  0.07406
##
## Coefficients:
##             Estimate Std. Error t value Pr(>|t|)
## (Intercept)  0.32936    0.02082   15.82   <2e-16 ***
## TSS         -0.50035    0.04517  -11.08   <2e-16 ***
## ---
## Signif. codes:  0 '***' 0.001 '**' 0.01 '*' 0.05 '.' 0.1 ' ' 1
##
## Residual standard error: 0.03708 on 998 degrees of freedom
## Multiple R-squared:  0.1095, Adjusted R-squared:  0.1086
## F-statistic: 122.7 on 1 and 998 DF,  p-value: < 2.2e-16
```

9.2 Broadstone Stream (taxonomic aggregation)

```
##
## Call:
## lm(formula = mean_err ~ TSS, data = mean_err_TSS)
##
## Residuals:
##       Min        1Q    Median        3Q       Max
## -0.042217 -0.007716  0.000558  0.006924  0.027255
```

```
##
## Coefficients:
##             Estimate Std. Error t value Pr(>|t|)
## (Intercept)  0.34058    0.00334  101.97   <2e-16 ***
## TSS         -0.36439    0.01151  -31.65   <2e-16 ***
## ---
## Signif. codes:  0 '***' 0.001 '**' 0.01 '*' 0.05 '.' 0.1 ' ' 1
##
## Residual standard error: 0.01135 on 998 degrees of freedom
## Multiple R-squared:  0.5009, Adjusted R-squared:  0.5004
## F-statistic:  1001 on 1 and 998 DF,  p-value: < 2.2e-16
```

9.3 Broadstone Stream (size aggregation)

```
##
## Call:
## lm(formula = mean_err ~ TSS, data = mean_err_TSS)
##
## Residuals:
##      Min       1Q   Median       3Q      Max
## -15.4077  -4.2887   0.1484   4.7925  13.4487
##
## Coefficients:
##             Estimate Std. Error t value Pr(>|t|)
## (Intercept)   73.714      5.097   14.46   <2e-16 ***
## TSS          -83.629      7.283  -11.48   <2e-16 ***
## ---
## Signif. codes:  0 '***' 0.001 '**' 0.01 '*' 0.05 '.' 0.1 ' ' 1
##
## Residual standard error: 5.828 on 998 degrees of freedom
## Multiple R-squared:  0.1167, Adjusted R-squared:  0.1158
## F-statistic: 131.9 on 1 and 998 DF,  p-value: < 2.2e-16
```

9.4 Broom

```
##
## Call:
## lm(formula = mean_err ~ TSS, data = mean_err_TSS)
##
## Residuals:
##      Min       1Q   Median       3Q      Max
## -0.24483 -0.03674  0.01409  0.04485  0.08370
##
## Coefficients:
##              Estimate Std. Error t value Pr(>|t|)
## (Intercept)  0.437254   0.005208   83.97  < 2e-16 ***
## TSS         -0.152599   0.020292   -7.52 1.22e-13 ***
## ---
## Signif. codes:  0 '***' 0.001 '**' 0.01 '*' 0.05 '.' 0.1 ' ' 1
##
## Residual standard error: 0.05713 on 998 degrees of freedom
## Multiple R-squared:  0.05363,    Adjusted R-squared:  0.05268
## F-statistic: 56.55 on 1 and 998 DF,  p-value: 1.218e-13
```

9.5 Capinteria

```
##
## Call:
## lm(formula = mean_err ~ TSS, data = mean_err_TSS)
##
## Residuals:
##      Min       1Q   Median       3Q      Max
## -0.21149 -0.07455  0.04157  0.07726  0.13695
##
## Coefficients:
##             Estimate Std. Error t value Pr(>|t|)
```

```
## (Intercept)  0.36582    0.01153  31.729  < 2e-16 ***
## TSS         -0.13714    0.02796  -4.906 1.09e-06 ***
## ---
## Signif. codes:  0 '***' 0.001 '**' 0.01 '*' 0.05 '.' 0.1 ' ' 1
##
## Residual standard error: 0.09871 on 998 degrees of freedom
## Multiple R-squared:  0.02354,    Adjusted R-squared:  0.02257
## F-statistic: 24.06 on 1 and 998 DF,  p-value: 1.087e-06
```

9.6 Caricaie Lakes

```
##
## Call:
## lm(formula = mean_err ~ TSS, data = mean_err_TSS)
##
## Residuals:
##      Min       1Q   Median       3Q      Max
## -0.22367 -0.04988  0.02288  0.06424  0.10604
##
## Coefficients:
##             Estimate Std. Error t value Pr(>|t|)
## (Intercept)  0.34378    0.00892  38.541  < 2e-16 ***
## TSS          0.28148    0.04888   5.758 1.13e-08 ***
## ---
## Signif. codes:  0 '***' 0.001 '**' 0.01 '*' 0.05 '.' 0.1 ' ' 1
##
## Residual standard error: 0.07685 on 998 degrees of freedom
## Multiple R-squared:  0.03216,    Adjusted R-squared:  0.03119
## F-statistic: 33.16 on 1 and 998 DF,  p-value: 1.131e-08
```

9.7 Grasslands

```
##
```

```
## Call:
## lm(formula = mean_err ~ TSS, data = mean_err_TSS)
##
## Residuals:
##       Min        1Q    Median        3Q       Max
## -0.113414 -0.045792  0.001642  0.041063  0.181017
##
## Coefficients:
##             Estimate Std. Error t value Pr(>|t|)
## (Intercept) 0.190539   0.007333  25.982  < 2e-16 ***
## TSS         0.373534   0.056238   6.642 5.08e-11 ***
## ---
## Signif. codes:  0 '***' 0.001 '**' 0.01 '*' 0.05 '.' 0.1 ' ' 1
##
## Residual standard error: 0.05478 on 998 degrees of freedom
## Multiple R-squared:  0.04233,    Adjusted R-squared:  0.04137
## F-statistic: 44.12 on 1 and 998 DF,  p-value: 5.076e-11
```

9.8 Mill Stream

```
##
## Call:
## lm(formula = mean_err ~ TSS, data = mean_err_TSS)
##
## Residuals:
##      Min       1Q   Median       3Q      Max
## -0.31085 -0.06180 -0.03214  0.11952  0.15870
##
## Coefficients:
##             Estimate Std. Error t value Pr(>|t|)
## (Intercept)  0.49738    0.01835   27.10   <2e-16 ***
## TSS         -0.42983    0.02642  -16.27   <2e-16 ***
## ---
```

```
## Signif. codes:  0 '***' 0.001 '**' 0.01 '*' 0.05 '.' 0.1 ' ' 1
##
## Residual standard error: 0.1155 on 998 degrees of freedom
## Multiple R-squared:  0.2096, Adjusted R-squared:  0.2088
## F-statistic: 264.6 on 1 and 998 DF,  p-value: < 2.2e-16
```

9.9 Small Reef

```
##
## Call:
## lm(formula = mean_err ~ TSS, data = mean_err_TSS)
##
## Residuals:
##       Min        1Q    Median        3Q       Max
## -0.142108 -0.058311  0.006842  0.057750  0.090019
##
## Coefficients:
##             Estimate Std. Error t value Pr(>|t|)
## (Intercept)  0.24598    0.01060  23.202   <2e-16 ***
## TSS         -0.02566    0.02490  -1.031    0.303
## ---
## Signif. codes:  0 '***' 0.001 '**' 0.01 '*' 0.05 '.' 0.1 ' ' 1
##
## Residual standard error: 0.06266 on 998 degrees of freedom
## Multiple R-squared:  0.001063,   Adjusted R-squared:  6.213e-05
## F-statistic: 1.062 on 1 and 998 DF,  p-value: 0.303
```

9.10 Tuesday Lake

```
##
## Call:
## lm(formula = mean_err ~ TSS, data = mean_err_TSS)
##
```

```
## Residuals:
##      Min       1Q   Median       3Q      Max
## -0.22724 -0.05970  0.02208  0.07776  0.10697
##
## Coefficients:
##             Estimate Std. Error t value Pr(>|t|)
## (Intercept)  0.44743    0.01961   22.82   <2e-16 ***
## TSS         -0.38757    0.03284  -11.80   <2e-16 ***
## ---
## Signif. codes:  0 '***' 0.001 '**' 0.01 '*' 0.05 '.' 0.1 ' ' 1
##
## Residual standard error: 0.08694 on 998 degrees of freedom
## Multiple R-squared:  0.1225, Adjusted R-squared:  0.1216
## F-statistic: 139.3 on 1 and 998 DF,  p-value: < 2.2e-16
```

9.11 Ythan

```
##
## Call:
## lm(formula = mean_err ~ TSS, data = mean_err_TSS)
##
## Residuals:
##      Min       1Q   Median       3Q      Max
## -15.4077  -4.2887   0.1484   4.7925  13.4487
##
## Coefficients:
##             Estimate Std. Error t value Pr(>|t|)
## (Intercept)   73.714      5.097   14.46   <2e-16 ***
## TSS          -83.629      7.283  -11.48   <2e-16 ***
## ---
## Signif. codes:  0 '***' 0.001 '**' 0.01 '*' 0.05 '.' 0.1 ' ' 1
##
## Residual standard error: 5.828 on 998 degrees of freedom
```

```
## Multiple R-squared:  0.1167, Adjusted R-squared:  0.1158
## F-statistic: 131.9 on 1 and 998 DF,  p-value: < 2.2e-16
```

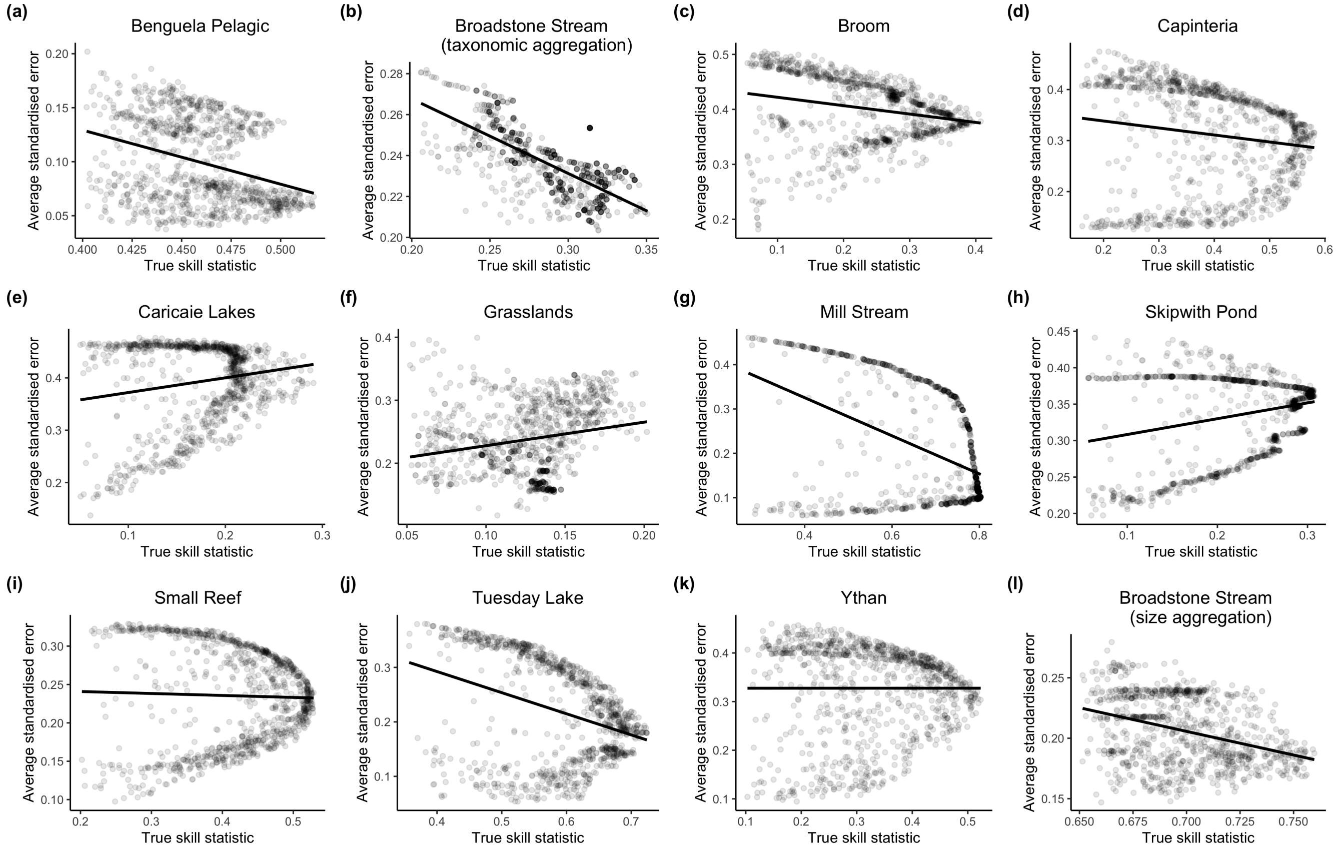

Figure S30: The mean standardised error of the food web properties predicted from the ADBM parameterised using rejection ABC plotted against the true skill statistic for each food webs. The solid lines are linear regressions (details in SI-S9).

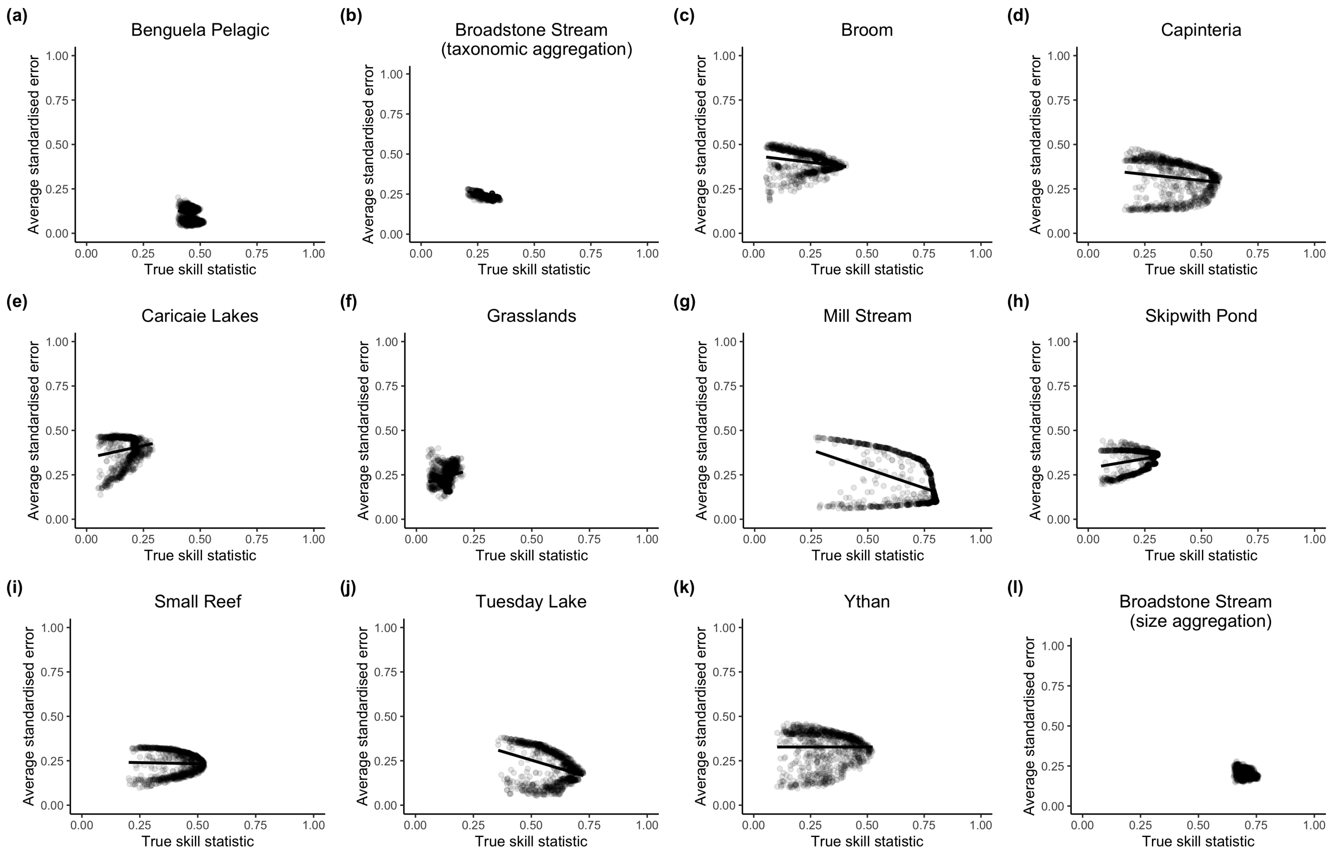

Figure S31: The mean standardised error of the food web properties predicted from the ADBM parameterised using rejection ABC plotted against the true skill statistic for each food webs. The solid lines are linear regressions (details in SI-S9). The limits of the horizontal and vertical axes have been set between 0 and 1.

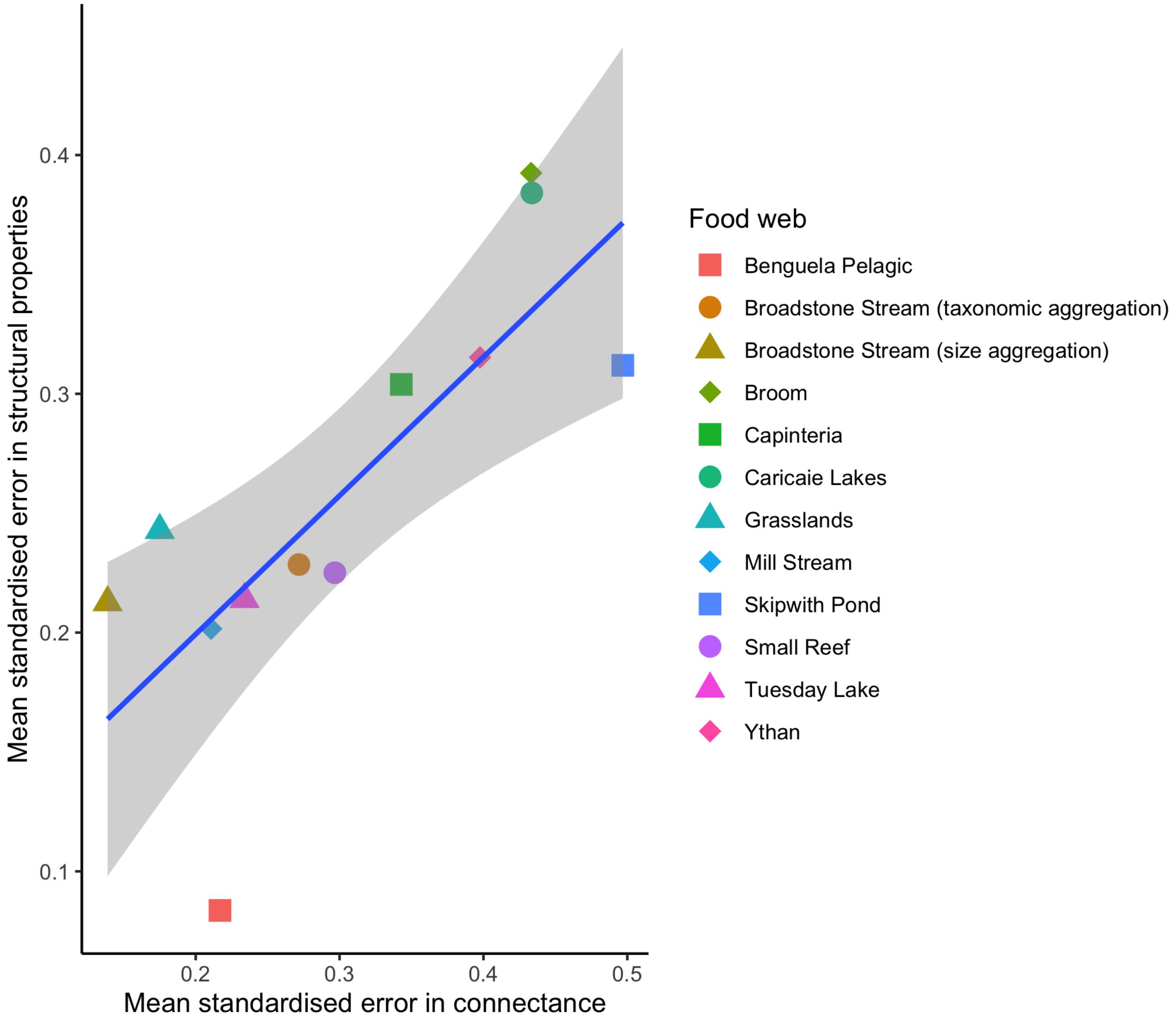

Figure S32: The mean standardised error in structural properties of food web versus mean standardised error in connectance. Solid blue line is linear regression ($t = 3.911$, $df = 10$, $P = 0.0029$)

References

Gelman, Andrew, and Donald B. Rubin. 1992. "Inference from Iterative Simulation Using Multiple Sequences." *Statistical Science* 7 (4): 457–72. https://doi.org/10.1214/ss/1177011136.

Marjoram, P., J. Molitor, V. Plagnol, and S. Tavare. 2003. "Markov Chain Monte Carlo Without Likelihoods." *Proceedings of the National Academy of Sciences* 100 (26): 15324–28. https://doi.org/10.1073/pnas.0306899100.
